# Mapping narratives on mental health to facilitate collaboration, communication and alignment of care: a systematic scoping review and interview study

**DOI:** 10.1136/bmjopen-2025-110663

**Published:** 2026-07-16

**Authors:** Deanne Spek, Marieke J J Ermers, Judith A C Rietjens, Megan M Milota, Niko J H Vegt

**Affiliations:** 1 Department of Design, Organisation and Strategy, Faculty of Industrial Design Engineering, Delft University of Technology, Delft, The Netherlands; 2 Department of Psychiatry, University Medical Centre Utrecht, Utrecht, The Netherlands; 3 Department of Public Health, Erasmus MC University Medical Center Rotterdam, Rotterdam, The Netherlands; 4 Department of the Julius Centre, University Medical Centre Utrecht, Utrecht, The Netherlands; 5 Department of Psychology, Education and Child Studies, Erasmus University Rotterdam, Rotterdam, The Netherlands; 6 Department of Human-Centred Design, Delft University of Technology, Faculty of Industrial Design Engineering, Delft, The Netherlands

**Keywords:** MENTAL HEALTH, PSYCHIATRY, PUBLIC HEALTH, Change management, Psychosocial Intervention, Social Support

## Abstract

**Abstract:**

**Objectives:**

We aimed to examine existing and emerging master narratives on mental health and to capture the changing emphasis in these narratives over time. Making these shifts explicit can facilitate alignment of actors and practices that must collaborate to support those seeking help.

**Design:**

We gathered data via a scoping review, semistructured interviews and podcast episodes. Data analysis consisted of a thematic analysis to identify narratives and a content analysis to map shifts in emphasis over time.

**Setting:**

Semistructured interviews and included podcast episodes were all Dutch. The scoping review included Dutch and English publications.

**Participants:**

Semistructured interviews were performed with 11 participants working in medical education and mental health provision. The selected podcast featured interviews with 15 people who aim to change the Dutch mental healthcare system.

**Results:**

We extracted four master narratives and observed a shift in emphasis as well as a coexistence of narratives. The narratives we found were: (1) treating a classification, (2) understanding the patient’s problem, (3) recovering life-balance and (4) building collective resilience. We found increasing emphasis on ‘building collective resilience’ when discussing the future of mental health.

**Conclusions:**

Our concretisation of narrative patterns underlying the perspectives and approaches in mental health is a first step in creating a collective understanding of the shifting master narratives, which enables working with it in practice. The shift has implications for mental health professionals, educators, policymakers and those seeking help. Future research should further examine these implications and explore the perspectives of individuals seeking mental health support and their network, both of which remain insufficiently addressed.

**STRENGTHS AND LIMITATIONS OF THIS STUDY**

- Data source, investigator and methodological triangulation of three data collection methods were used to ensure reliability and validity of the findings.
- For the scoping review, a systematic search and screening process were performed.
- The addition of a podcast analysis allowed for capturing broader views in society on mental health.
- Interviewees were predominantly recruited from one institution, and podcast episodes were limited to Dutch speakers.

## Introduction

 We are witnessing a change in attitudes, perspectives and views with regards to mental health. Where mental health was previously approached as a disorder in a single human, it is increasingly also seen as existing in interaction with others and society.^1–4^ Biological and psychological dimensions of mental health are extended with existential, social, cultural, environmental and political dimensions.^1 3 5^ This extended understanding of mental health requires a change in how professionals and those seeking help interact^1 6^ as well as alignment of and collaboration between professionals of different domains to support those seeking help.^2 7^ Making explicit the shift in understandings of mental health can facilitate alignment of all actors and practices that are part of the mental health system; it can support conversations and expectations in the consultation room, fuel adaptations in medical education to better prepare professionals of the future and foster necessary changes in regulatory and financial frameworks.^4 8 9^

Others have investigated shifts in discourses on mental health.^10–16^ However, discourse research often addresses the linguistic component of mental healthcare interactions (the words that are used to refer to people seeking help and mental health/illness itself). No overview exists of how attitudes, perspectives and approaches with regards to mental health are shifting. One means of creating such an overview is to map the shift of master narratives on mental health. Master narratives are culturally shared stories that guide thoughts, beliefs, values and behaviours.^17–19^ They are broad, culture-specific stories that are represented in various aspects of a society, such as legal frameworks, popular language and practices, perceived social roles and media representations.^17^ Master narratives on mental health may thus be reflected in how people talk about mental health, in their expectations of mental health services, or in responses by healthcare professionals towards those seeking help. Capturing shifts in emphasis in different master narratives can help us better understand changing beliefs, values and behaviours and in turn support alignment of expectations, services and regulations.

In reality, a strict distinction in narratives does not exist and master narratives gradually shift over time.^17 20^ Demarcating clear boundaries to capture a shift would entail constructing an artificial, simplified representation of reality. Furthermore, master narratives do not provide insight into how individuals weave various stories, themes and modalities into their developing normative stance towards mental health. However, the simplification is a first step in creating a collective understanding of shifting master narratives, which enables working with it in practice.^20^ Therefore, we aimed to answer the following questions: (1) What are the existing and emerging master narratives on mental health? (2) How has the emphasis in these narratives shifted over time?

Since master narratives are context-sensitive,^17 19^ we focused on those in the Euro-American context, paying particular attention to those in Dutch society. Historical understandings of mental health in the Dutch society have been extensively studied.^21 22^ Mental health has been framed politically as a moral disorder, as an illness to be treated in the hospital bed, and as a biological problem driven by the pharmaceutical industry.^21^ Dominant narratives on mental health evolved together with changing societal and policy contexts. To capture presently dominant and possible future master narratives, we, therefore, moved beyond what is observed in scientific literature and added the views and practices that are shared in everyday life.^20^

## Methods

### Data collection

To get an understanding of something as manifold as master narratives, we used investigator, data source and methodological triangulation.^23 24^ This approach of gathering data via multiple methods allowed us to discover ‘meaningful information that may have remained undiscovered with the use of only one approach or data collection technique in the study’.^25^ We gathered data on evolving narratives via a systematic scoping review, semistructured interviews and review of a podcast series consisting of interviews with changemakers in Dutch mental healthcare.

#### Systematic scoping review

The systematic scoping review followed the five stages as outlined by Arksey and O'Malley^26^: (1) defining the research question, (2) identifying relevant studies, (3) study selection, (4) charting the data and (5) collating, summarising and reporting the results. Steps 4 and 5 were performed with data from the scoping review, semistructured interviews and podcast interviews combined and are described below.

We searched in SCOPUS, WorldCat and PubMed on 4 August 2023. Search terms were generated from the research question and included the following areas of interest: (a) mental health and related areas such as psychology and psychiatry, (b) terms referring to a shift or change and (c) terms referring to expressions of narratives such as attitudes or beliefs. This search was updated on 2 May 2026, following the same process as described below, and updated by adding the terms ‘discourse’ and ‘narrative’ as terms that could refer to a shift for the full period of 2018–2026 (see online supplemental materials 1 for the full search strategies).

Two reviewers (NJHV and DS) screened all titles and abstracts to identify relevant articles using the following inclusion criteria: (a) published after 2018 to gather contemporary views, (b) describing narratives on mental health (ie, attitudes, perspectives or other expressions of narratives on mental health), (c) written in Dutch or English and (d) full-text availability. In line with the scoping review guidelines,^26 27^ quality appraisal of included studies was not undertaken since this study aims to capture master narratives in a wide range of resources even including podcast interviews. Disagreements regarding inclusion were discussed until consensus was reached.

**Figure 1:**
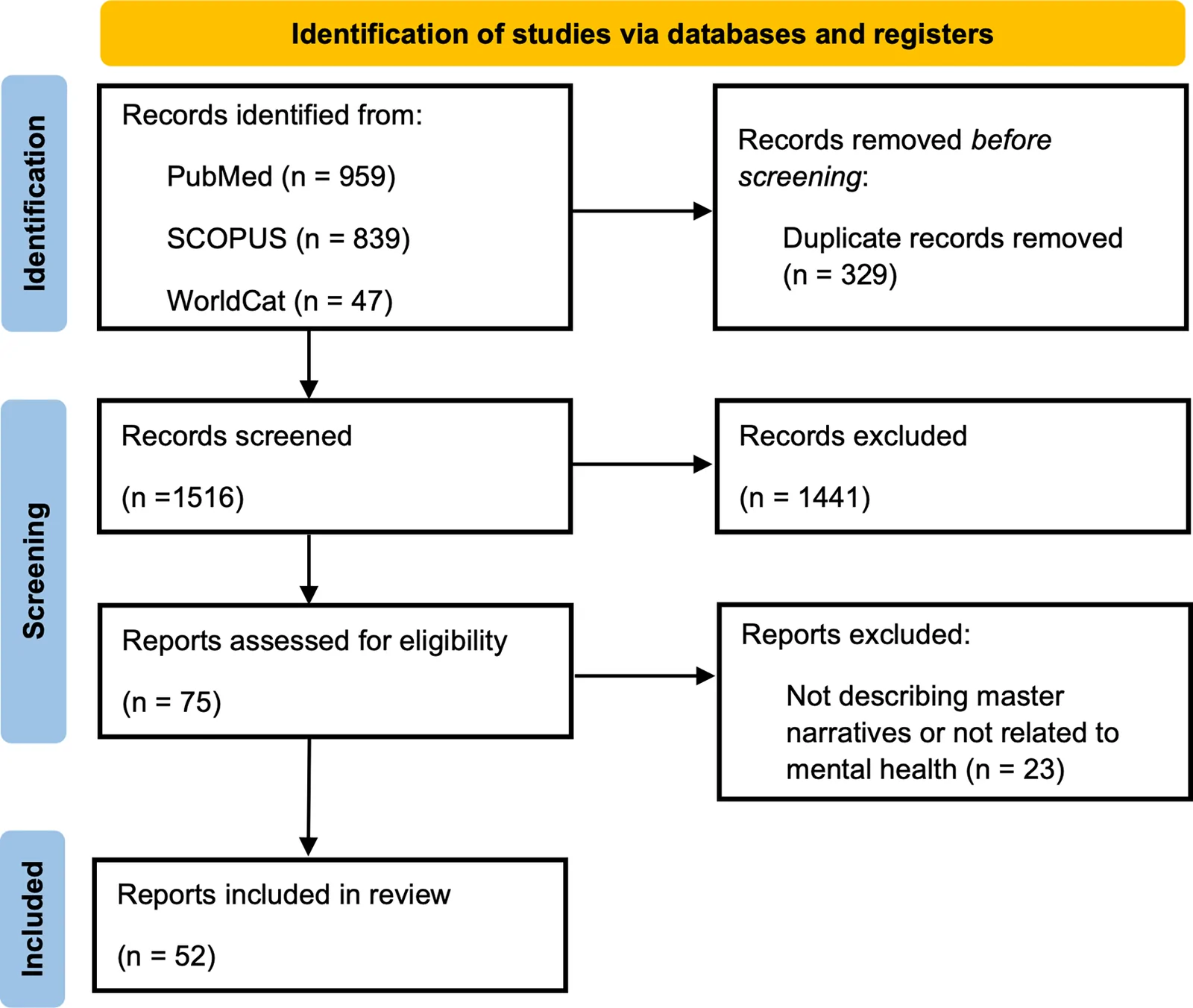
PRISMA flow diagram of systematic scoping review. PRISMA, Preferred Reporting Items for Systematic Reviews and Meta-Analyses.

The full-text publications were subsequently assessed for eligibility. We excluded publications because they addressed somatic rather than mental health or did not describe representations of narratives. For example, some abstracts mentioned overall approaches in mental healthcare, but the article itself only focused on a specific intervention without reflecting on the overall narrative. The Preferred Reporting Items for Systematic Reviews and Meta-Analyses (PRISMA) diagram for the collection of literature is presented in figure 1.^28^

#### Semistructured interviews

The semistructured interviews were conducted by DS, a female design graduate and junior researcher with experience in interviewing and qualitative research at the time of the interviews, in the context of a project on redesigning medical education at the University Medical Centre Utrecht (UMCU). The aim of the interviews was to gather interviewees’ perspectives on how the approach to mental health is and should be changing in society and healthcare, followed by their suggestions for medical education based on these perspectives. The interview guide can be found in online supplemental materials 1.

Because master narratives are largely invisible except for those who deviate from it,^17^ people known for deviating from the dominant approach in psychiatry were interviewed. These people were identified with support from medical and educational staff by asking them which people at UMCU ‘do or think differently’. This resulted in nine UMCU interviewees and was complemented with two interviewees from a broader professional network (table 1). Participants were recruited via mail, and interviews were conducted via Microsoft Teams or in person. Interviews took 60–90 min and started with verbal informed consent. The interviews were audio-recorded and transcribed verbatim. Interviewees’ opinions were examined to see if findings from literature reflect what is perceived in everyday practice and to gain more insight into how master narratives might evolve in the future.

**Table 1:** Interviewees of semistructured interviews and podcast episodes

	Semistructured interviews (n=11)	Interviews in podcast episodes (n=15)
Gender Female Male	5 (45%) 6 (55%)	7 (47%) 8 (53%)
Role in mental healthcare Psychiatrist Psychologist Family therapist Expert-by-experience Other	4 (36%) 1 (9%) 1 (9%) 1 (9%) 4 (36%)	5 (33%) 2 (13%) 1 (7%) 4 (27%) 3 (20%)
Role in mental health research/education Student Professor Coordinator/creator of education Teacher None	2 (18%) 2 (18%) 3 (27%) 3 (27%) 1 (9%)	0 4 (27%) 1 (7%) 1 (7%) 9 (60%)
Explicitly using personal experiences in work with others Yes No	2 (18%) 9 (82%)	5 (33%) 10 (67%)

#### Podcast interviews

To broaden our understanding and to ensure professional perspectives were not only UMCU-specific, we included all 11 episodes from the podcast ‘Hoe de GGZ verandert’ (How mental healthcare is changing) created in 2020 by Carlijn Welten.^29^ Podcasts are considered representative for public views^30^ and increasingly used in academic research.^31^ Furthermore, ‘Hoe de GGZ verandert’ presents 15 diverse perspectives of people who position themselves as deviating from the dominant understanding of mental health (table 1). To address methodological considerations around the use of podcasts, Kulkov *et al*^31^ propose four main selection criteria. ‘Hoe de GGZ verandert’ achieved the maximum score on all four as it (1) strongly aligns with our research question, (2) is credible, (3) receives high ratings on different platforms (including Spotify and Apple podcasts) and (4) is freely accessible for verification. Because of this accessibility, we deemed it unnecessary to ask all podcast interviewees for consent. We did ask the creator of the podcast for consent after showing her how we had used and referred to her podcast episodes.^30 31^ Data were extracted from the episodes via relistening, noting down timestamps of relevant fragments and transcribing these verbatim in line with Kulkov *et al*’s methodological recommendations for podcasts.^31^

One person interviewed by Welten was also included in our semistructured interviews. The podcast focused mainly on how mental healthcare is changing; we were also interested in current narratives and in implications for medical education. The podcast data and semistructured interview data from this person were treated as two separate sources to rightfully represent what narratives were brought forward by podcast data versus semistructured interview data. Furthermore, the podcast interview took place 2 years prior to the semistructured interview.

### Data analysis

The data were analysed using two methods regularly used in extracting master narratives.^20 32^ With an inductive thematic analysis, we extracted the narratives present in the data,^33^ using open, axial and selective coding.^34^ Via a deductive content analysis, we explored how the emphasis on different narratives shifted over time.^35^

#### Thematic analysis

The thematic analysis followed the steps of Braun and Clarke,^33^ using Microsoft Excel. After familiarisation, open coding of the full-text articles, podcast interviews and semistructured interview transcripts was performed to identify text units that refer to narratives on mental health. We used the full article and not only the results, as all parts of a publication may be relevant for this study. Next, axial coding was conducted by iteratively clustering codes into themes. Finally, selective coding took place to define the narratives on mental health expressed by these themes. Two researchers (DS and NJHV) met after each step to assess each other’s results and reach consensus. The resulting codes, themes and narratives were also discussed with the team to evaluate if this structure resonated with what is experienced in the field (MJJE) and was methodologically sound (JACR).

#### Content analysis

Quantifying the coding with a content analysis^35^ allowed for the discovery of a possible shift in the narratives. All text units resulting from the thematic analysis were deductively coded with respect to the timeframe they represented. Data were coded with ‘present’ when it contained views, observations or opinions marked as contemporary or expressed in present tense (2018–2026); as ‘past’ when it referred to views, observations or opinions before the publication, interview or podcast; and as ‘future’ when they referred to prospective or aspirational views. We then counted the number of sources mentioning the past, present or future within each narrative to create a heatmap (table 2). Such a visual representation in which data values are represented by colour intensity allowed us to gain an intuitive understanding of emphasis on different narratives.

**Table 2:** Percentage of sources that contain text units about one of the narratives in the past, present or future

*Narratives*	Past	Present	Future
Treating a classification	22%	64%	3%
Understanding the patient’s problem	5%	50%	40%
Recovering life-balance	5%	44%	49%
Building collective resilience	9%	53%	68%

Combining three forms of collected data not only enabled a richer understanding of master narratives in mental health^36^ but also introduced challenges. To avoid privileging the richness of interview and podcast data,^23^ one author (NJHV) did not listen to the interviews or podcasts but started with the scoping review. To address the fact that academic literature had already gone through interpretation of the authors and provided more explicit indications of master narratives, particular attention was given to the coding of interview and podcast data. Finally, analysis of data extracted from all three sources was combined to capture the totality of master narratives present. After completing the analysis, we examined how the different narratives were represented by the three data sources.

## Results

From the 78 publications and (podcast) interview transcripts, we extracted four narratives and found a shift in emphasis among these narratives over time. These findings were consistent across all three data types as well as across multiple sources. Table 2 shows the distribution of the data along with examples of the analytical procedure. A detailed overview of codes, themes and narratives per source is provided in online supplemental materials 1. All text units with codes, themes and narratives can befound here: https://doi.org/10.5064/F6E1OMLI.^37^

**Table 3:** Summary of findings: the distribution of the data and the analytical procedure

Source	Text unit extracted	Timeframe	Code	Theme	Narrative
**78 sources**	**859 text units**	**3 timeframes**	**35 codes**	**12 themes**	**4 narratives**
Distribution over data types					
52 articles	533	3	35	12	4
11 semistructured interviews	271	3	33	12	4
15 podcast episodes	55	3	21	12	4
Examples analytical procedure					
Episode 8, female	‘Normalizing is accepting variety, seeing the power of the exception. You need exceptions of certain traits to move forward as a society.’	Future	Creating collective strength	Collective health and acceptation	Building collective resilience
Stolper *et al*^53^	‘The still dominant medical model in which mental disorders are conceptualized as a disease of the individual that should be treated as such.’	Present	Problem of individual	Individual who needs help	Understanding the patient’s problem
Interviewee 7, male	‘We really have had that brain fetishism.’	Past	Addressing the brain and biology	Classification-specific treatment	Treating a classification

**Figure 2:**
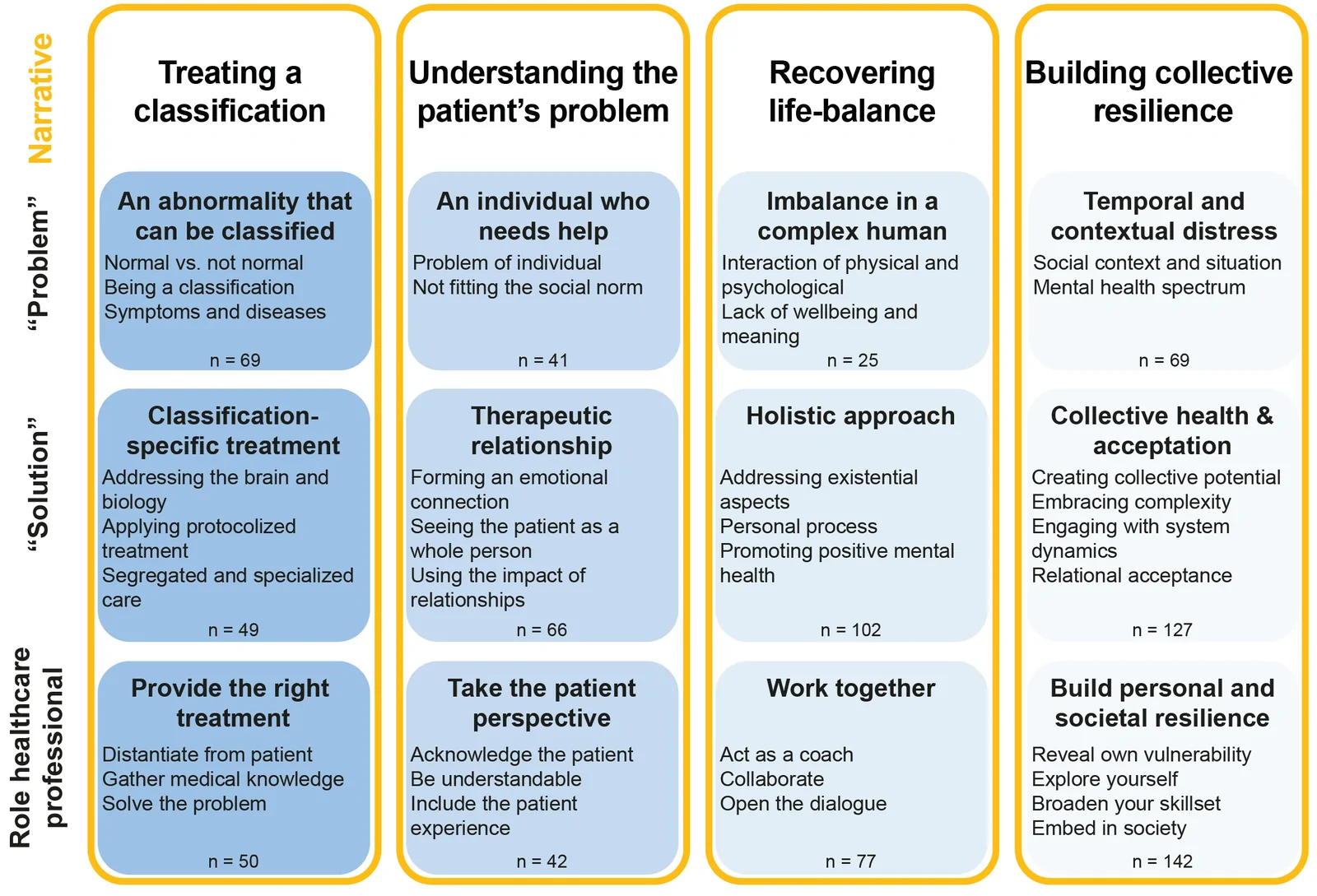
The four master narratives (top) with the themes (in bold) and codes they encompass, including the number of text units in each theme.

We will first describe the four narratives: (1) treating a classification, (2) understanding the patient’s problem, (3) recovering life-balance and (4) building collective resilience. These narratives with their themes and corresponding codes are summarised in figure 2. As the figure shows, the themes address three aspects of each narrative. The first aspect is how the aetiology of mental health is understood: what is seen as the ‘problem’. The second aspect refers to desired actions to alleviate suffering or (re)gain mental health: what is seen as the ‘solution’. The third aspect concerns what is expected from the mental health professional in this process. The descriptions are supported by table 4, showing one of the text units representing the ‘problem’, ‘solution’ and professional role in each narrative. We end with discussing the heatmap created to see how emphasis on the narratives has shifted over time.

**Table 4:** Text units coded and clustered into the themes representing the 'problem’, 'solution’ and role of the professional for each of the four narratives

Narrative	Treating a classification	Understanding the patient’s problem	Recovering life-balance	Building collective resilience
'Problem’	An abnormality that can be classified ‘All your behavior is judged based on the diagnosis (this is your borderline). As a result, you kind of lose your identity: what is you and what is your disorder?’ (podcast interviewee 8, female)	An individual who needs help ‘When you assume that life is malleable, something is wrong with the individual when it does not work out.’ (podcast interviewee 10, female)	An imbalance in a complex human ‘Health is about the ability to adjust to and manage medical, social and mental challenges in order to pursue life goals that are meaningful to the person(…)restoration of health is not the goal, but rather the means to enable the patient to find and pursue meaningful goals.’ (article of van Os *et al*^54^, p.92)	Temporal and contextual distress ‘I think we start to look at it as a spectrum from healthy, feeling good, having good relationships and being able to develop … to having some stress but being able to deal with it … to not feeling well but being able to manage it with some time off and a few good conversations … to moments when you think: when this goes on for long, something will happen with me … to moments you can’t do it anymore, you feel bad and trouble others with it as well.’ (interviewee 10, male)
'Solution’	Classification-specific treatment ‘When you use a more medical model to look at psychological complaints, it quickly became: you have a complaint, which disorder belongs to it? And when the disorder is found, a certain treatment belongs to it.’ (interviewee 3, female)	Therapeutic relationship ‘Available research involving service users’ perceptions raises questions about the nature of power in provider-user encounters and stresses the emphasis on relational work.’ (article of Karbouniaris *et al*^55^, p.25)	Holistic approach ‘The impact of suffering causes someone to reinvent himself and adjust his goals. […] Recovery coaches, experts-by-experience and recovery colleges rapidly appear. Now they are separated from the mental health system, but let’s collaborate.’ (podcast interviewee 1, male)	Collective health and acceptation ‘Support each other. One person’s power is here, another’s there. Then we help each other, need each other and feel that as a society. That it becomes not only a collective responsibility, but a collective power.’ (interviewee 6, female)
Role professional	Provide the right treatment ‘Society expects mental health care to solve problems and current Dutch mental health care is also set up this way at the moment.’ (article of Lorenz-Artz *et al*^38^, p.6)	Take the patient perspective ‘The 5 minutes you have really are the 5 minutes for them [patients], while for a physician or psychiatrist it is only one of the many conversations on the day. So, make sure the patient is seen and heard and can share his story.’ (interviewee 8, male)	Work together ‘I have a lot of knowledge, you have lots of knowledge about yourself. Together we come to a story […] and from there we identify mechanisms in how we can help you break the patterns which got you stuck.’ (interviewee 7, male)	Build personal and societal resilience ‘We [medical students] have chosen a study in which you not only study, but also have to be aware of society and really be a human being.’ (interviewee 8, male)

### Treating a classification

In the first master narrative, the perceived problem was depicted as an abnormality that could be classified. A distinction was made between normal and abnormal or between those with and without a mental illness. This distinction was based on the presence of symptoms and captured in a classification. According to the data, the language and way these classifications are used can lead to patients identifying themselves with a classification and being identified with it by others.

According to our data, this narrative proclaims that a classification is best addressed by classification-specific treatment in line with evidence-based protocols. According to both the literature and interviewees, such treatments are best provided by specialised mental healthcare professionals (MHCPs). Furthermore, the sources explained that distinguishable classifications allow for (neuro)biological research on these classifications.

Hence, in this narrative, MHCPs are expected to solve the problem using their medical knowledge. The literature and interviewees described how MHCPs working in line with the narrative of ‘treating a classification’ are required to stay professional, remain in control and maintain professional distance from those with the classification.

### Understanding the patient’s problem

The second master narrative depicts the problem as individuals needing help. The focus on the individual shifts the problem from an objective classification to a person with problems or deficits.

The necessary support in this narrative was characterised by a therapeutic relationship. Authors and (podcast) interviewees described that a therapeutic relationship starts with seeing the patient as a person, listening and making a real connection. The human relationship that is created in this way was seen as the prerequisite to recovery.

The act of relationship-building requires the MHCP to take the perspective of the patient. Both literature and interviewees explained the necessity of gaining experience with and developing openness to the patient experience. Furthermore, they emphasised that MHCPs in this narrative should acknowledge the vulnerability of the patient. The data suggested that MHCPs should be understandable, tailoring their pace and language to each patient.

### Recovering life-balance

In the third master narrative, the problem could be summarised as imbalances in a complex human. Imbalance and complexity were represented in two ways in the data. The first was an imbalance in physical and psychological processes that are mutually influencing each other. The second was an imbalance in meaning and self-understanding due to the disruptive effect of mental health issues.

The data indicated that addressing this complexity and imbalance requires a holistic approach. A holistic approach meant covering existential aspects and attuning to the personal process of recovery. Existential aspects included reflecting on who you are, realigning your life story, self-acceptance and learning to relate to a new version of yourself. Literature and interviewees also described that recovery moves beyond alleviation of suffering. In this narrative, professionals should promote positive mental health and help those who seek help in building resilience for the future.

Within the process of ‘recovering life-balance’, the MHCP is expected to act as a coach and open the dialogue according to the literature and (podcast) interviewees. Together, the person seeking help, their network and the MHCP develop an understanding of the complex situation. In that sense, the person seeking help leads the recovery process with the MHCP nudging movement in the right direction. Literature and (podcast) interviewees also stated that the MHCP should seek collaboration with others in the healthcare domain, such as experts-by-experience.

### Building collective resilience

In the final master narrative, the predominant problem was described as temporal and contextual distress. Mental health was conceptualised as a spectrum on which everyone moves over time, depending on their context and situation. The contextual aspect of (family) culture was especially seen as influencing mental health.

Due to movement along a spectrum, good mental health is created via collective health and acceptance in society. Collective health could be supported by creating collective strength. With collective, literature and interviews referred to both the family as a collective as well as prevention and community approaches. In the narrative of building collective resilience, professionals must engage with the dynamics of social and environmental systems instead of focusing on the individual. However, various authors and interviewees explained that a systemic approach exists alongside a more individual and biological approach. To address temporal and contextual distress, one must embrace the complexity of multiple ‘problems’ and ‘solutions’ interacting with each other. Finally, individual and collective acceptance was described in the literature and interviews as a relational process. Feeling accepted by those around you or peers in group support was a catalyst for personal acceptance and meaning-making according to the literature and interviewees.

Building collective resilience requires MHCPs to embed themselves in society, which was referred to by literature and interviewees as taking a societal perspective as well as collaborating with other domains, such as education. Furthermore, the data showed that in this narrative MHCPs need to broaden their skillset with cultural skills, skills that allow self-reflexivity, skills in explaining complexity and skills in knowing when to apply which approach. For the development of such skills, it was deemed necessary that MHCPs explore themselves: their own professional stance and personal complexity. Being open and vulnerable about oneself and the limited knowledge one has is expected in this narrative.

### Shifting emphasis on narratives

To discover how the emphasis on these narratives shifts over time, the number of sources mentioning the past, present or future within each narrative were counted. The resulting heatmap is shown in table 2. This heatmap shows that hardly any sources mentioned ‘treating a classification’ as a desired perspective in the future (only by 3% of the sources). Instead, it was mostly mentioned as the main emphasis in the past (22%) and especially present (64%). The narratives of ‘understanding the patient’s problem’ and ‘recovering life-balance’ were mainly mentioned in relation to the present (by 50% and 44% of the sources, respectively) as well as something future-oriented (40% and 49%). ‘Building collective resilience’ was described by many sources as narratives in the present (53%) and was particularly emphasised in the future (68%). The heatmap shows that the data do not suggest an evolution from one narrative into another, but instead a broadening of the overall perspective on mental health to include societal origins and responsibilities.

## Discussion

Our understanding of mental health is changing. In this article, we aimed to systematically map the shift in emphasis on various master narratives, using input from academic literature and (podcast) interviews with people who work on changing the mental healthcare system. This concretisation is a first step in creating a collective understanding of the shifting master narratives, which enables working with it in practice. We extracted four master narratives on mental health: (1) treating a classification, (2) understanding the patient’s problem, (3) recovering life-balance and (4) building collective resilience. Our analysis showed a shift in emphasis towards narratives of ‘understanding the patient’s problem’, ‘recovering life-balance’ and especially to ‘building collective resilience’. For the future mental health system, a coalescence of narratives into an increasingly complex understanding of mental health thus seems to be occurring.

Our findings align with the rise of publications and practices on person-centred and recovery-oriented mental health support,^5 6 38 39^ and the increasing emphasis on contextual and relational factors contributing to mental health issues.^1 3 40 41^ Specifically, the coexistence of biomedical with social, contextual and recovery-oriented discourses has been emphasised in accounts of individual experiences^42^ and in studies on changing discourses in mental health.^11 14^ By moving beyond the ‘now’, this study adds knowledge on what approach and perspective on mental health we might be moving to: a narrative conceptualising mental health as temporally and contextually situated, emphasising the necessity of personal, collective and societal resilience alongside genetic and biological factors. Such knowledge can help professionals, educators, policymakers and all others involved in aligning perspectives and defining necessary steps to support such a future.

### Implications

The shifting master narratives require MHCPs to combine and negotiate different understandings when providing care, since different master narratives enable different ways of responding and understanding.^17 19^ Without awareness or training, not only MHCPs but professionals working in all layers of the system are susceptible to rigidity in their approaches or discourses that result from adhering unreflexively to a dominant master narrative.^10^ The findings of this study could be used as inspiration for the development of awareness, yet future research is needed to determine how this can best be achieved.

The shifting master narratives also have direct implications for the distribution and level of agency given to various actors in a given care process. In line with a review on the concept of person-centred care,^43^ we found a continuum of approaches from taking the perspective of the person with mental health needs, to sharing control together, to placing more control in the hands of the person and their network. Tools to give the person and their network more agency are, for example, developed by Veldmeijer and colleagues.^44^ Yet, the narrative of ‘building collective resilience’ even argues that responsibility should be shared by the general society. Awareness of the shifting narratives can facilitate alignment of these interactions and roles and supports the creation of mutual understanding, which is key to collaboration in mental health.^45–47^ Future research should look into the reconfiguration of roles that accompany the shifting narratives on mental health, especially on what is needed to support help seekers, their networks, the professionals involved and the broader society to give form to these roles in practice.

The narrative of ‘building collective resilience’ furthermore implies a greater role for MHCPs in society and for society in mental health support. This in turn requires collaboration and alignment of task divisions, financial systems and regulatory bodies. Experiments with such collaboration and alignment are already taking place. For example, van den Broek and colleagues^48^ describe how a provincial governing body, mental health institutions and health insurance providers aligned to provide new forms of mental health support in one Dutch community. Furthermore, in trials with improving Ecosystems of Mental Health new collaborations across domains are explored.^7^ However, it is still a challenge to extrapolate these pilots and provide integrated mental health support in everyday practice.

The implications discussed above—moving between different ways of understanding, shifting responsibilities, taking a societal role and collaborating with different layers of the mental health system—are all important topics to address in medical education as well. Although education was not a specific focus of this study, several of the sources suggest that current medical education falls behind in teaching the skills necessary for the future.^49 50^ Specifically, the narrative of ‘building collective resilience’ proposes supporting students in developing their skills and professional position in society. While we advise education to address this societal role and other aspects of the narrative ‘building collective resilience’, this study showed that the future requires navigating between master narratives. This implies the necessity for education to foster the development of skills when addressing existential aspects, skills in relationship-building and knowledge on diseases and disorders. The concretisation of shifting emphasis on narratives can help in collaboration, communication and alignment on educational development, just as it can serve this role in the other parts of the mental health system.

### Limitations and future research

First of all, master narratives are a useful tool to capture what is going on in mental health, but they are also unidimensional conceptualisations of how we weave various stories, themes and modalities into our developing normative stance towards mental health in everyday life. As individuals, we use different aspects of master narratives to define our own narrative.^20^ This is a nuanced, individual and ongoing negotiation. We recommend further research into the negotiation between personal narratives and the shifting master narratives, and how this affects the interaction with MHCP and other professionals.

The second limitation is the predominantly UMCU-based interviewees. We balanced this by including podcast interviews with people from all over the country, but only the academic literature includes views from outside the Netherlands. However, triangulation^23 24^ of the different data types showed us that findings were consistent across all three data sources, thus providing an indication for generalisability to Euro-American countries. Future research could expand beyond Euro-American cultures, as different cultures have different understandings of health^51^ and thus of mental health.

Third, the representation of patients’ perspectives is limited in the included data. Four academic articles were based on patient perspectives.^46 51–53^ Only 1 of the 11 interviewees and 4 of the 15 podcast interviewees were experts-by-experience. We, therefore, propose future research to elaborate on the narratives on mental health specifically from the perspective of those who seek help and their network.

Finally, our methods primarily emphasised the future. The semistructured interviews did address the present, but the context of the interview and a large part of the questions were about what should change as we move into the future. However, by explicating such changes, current narratives are often implicitly recognised as existing and continuing to exist.^17^ Authors of the academic literature and (podcast) interviewees may not have mentioned the ‘treating a classification’ narrative frequently when referring to the future because they assume this will continue existing in the future. Furthermore, the eligibility assessment of articles from the scoping review showed that articles which take a biomedical view as the starting point were often not included. These articles did not elaborate on this narrative or how it would remain to exist in the future. Authors of these articles may have taken this perspective on mental health for granted.

## Conclusion

This study presents a first overview of how master narratives on mental health are shifting towards a more social understanding of mental health. This overview can already serve as a tool for conversations, sharing perspectives and discussing possibilities for the ongoing transformation of the mental health system. Thereby, the findings of this study can promote alignment between different professionals of the mental healthcare system and beyond as well as regulatory bodies when coordinating mental healthcare provision for the future.

## Supplementary material

10.1136/bmjopen-2025-110663 online supplemental file 1

## Data Availability

Data are available in a public, open access repository.
