# Supplementary material for: Mapping narratives on mental health to facilitate collaboration, communication and alignment of care: a systematic scoping review and interview study
Source: BMJ Open. 2026 Jul 16;16(7):e110663. doi: 10.1136/bmjopen-2025-110663 (PMC13384187; doi:10.1136/bmjopen-2025-110663)
Supplement: online supplemental file 1 [file bmjopen-16-7-s001.pdf]

Supplementary Materials: Mapping Narratives on Mental Health to Facilitate Collaboration, Communication and Alignment of Care: A Systematic Scoping Review and Interview Study

SUPPLEMENTARY MATERIALS 1: SEARCH STRATEGIES FOR EACH DATABASE

SCOPUS (August 4, 2023)

TITLE-ABS-KEY(("expectation" OR "trend" OR "direction" OR "approach" OR "attitude" OR "view" OR "transition" OR "shift" OR "perspective") AND ("mental health*" OR "mental wellbeing" OR "psychological" OR "psychiatry") AND ("dutch" OR "Netherland*")) AND (LIMIT-TO (PUBYEAR,2023) OR LIMIT-TO (PUBYEAR,2022) OR LIMIT-TO (PUBYEAR,2021) OR LIMIT-TO (PUBYEAR,2020) OR LIMIT-TO (PUBYEAR,2019) OR LIMIT-TO (PUBYEAR,2018)) AND (LIMIT-TO (LANGUAGE,"English") OR LIMIT-TO (LANGUAGE,"Dutch")) AND (LIMIT-TO (AFFILCOUNTRY,"Netherlands")) AND (LIMIT-TO (SUBJAREA,"PSYC") OR LIMIT-TO (SUBJAREA,"SOCI") OR LIMIT-TO (SUBJAREA,"ARTS") OR LIMIT-TO (SUBJAREA,"MULT"))

WorldCat (August 4, 2023)

Ab("expectation" OR "trend" OR "direction" OR "approach" OR "attitude" OR "view" OR "transition" OR "shift" OR "perspective") AND Ti("mental health*" OR "mental wellbeing" OR "psychological" OR "psychiatry") AND Ti("dutch" OR "Netherland*") AND (yr:2018)
Held By Library TU Delft checked

PubMed (August 4, 2023)

((("expectation"[Title/Abstract] OR "trend"[Title/Abstract] OR "direction"[Title/Abstract] OR "approach"[Title/Abstract] OR "attitude"[Title/Abstract] OR "view"[Title/Abstract] OR "transition"[Title/Abstract] OR "shift"[Title/Abstract] OR "perspective"[Title/Abstract]) AND ("mental health*"[Title/Abstract] OR "mental wellbeing"[Title/Abstract] OR "psychological"[Title/Abstract] OR "psychiatry"[Title/Abstract]) AND ("dutch"[Title/Abstract] OR "Netherland*"[Title/Abstract]))) AND (("2018"[Date - Publication] : "3000"[Date - Publication])) AND (Dutch[Language] OR English[Language])

SCOPUS (May 2, 2026)

TITLE-ABS-KEY(("expectation" OR "trend" OR "direction" OR "approach" OR "attitude" OR "view" OR "transition" OR "shift" OR "perspective") AND ("mental health*" OR "mental wellbeing" OR "psychological" OR "psychiatry") AND ("dutch" OR "Netherland*")) AND (LIMIT-TO (PUBYEAR,2026) OR LIMIT-TO (PUBYEAR,2025) OR LIMIT-TO (PUBYEAR,2024) OR LIMIT-TO (PUBYEAR,2023)) AND (LIMIT-TO (LANGUAGE,"English") OR LIMIT-TO (LANGUAGE,"Dutch")) AND (LIMIT-TO (AFFILCOUNTRY,"Netherlands")) AND (LIMIT-TO (SUBJAREA,"PSYC") OR LIMIT-TO (SUBJAREA,"SOCI") OR LIMIT-TO (SUBJAREA,"ARTS") OR LIMIT-TO (SUBJAREA,"MULT"))

TITLE-ABS-KEY(("narrative" OR "discourse") AND ("mental health*" OR "mental wellbeing" OR "psychological" OR "psychiatry") AND ("dutch" OR "Netherland*")) AND (LIMIT-TO (PUBYEAR,2026) OR LIMIT-TO (PUBYEAR,2025) OR LIMIT-TO (PUBYEAR,2024) OR LIMIT-TO (PUBYEAR,2023) OR LIMIT-TO (PUBYEAR,2022) OR LIMIT-TO (PUBYEAR,2021) OR LIMIT-TO (PUBYEAR,2020) OR LIMIT-TO (PUBYEAR,2019) OR LIMIT-TO (PUBYEAR,2018)) AND (LIMIT-TO (LANGUAGE,"English") OR LIMIT-TO (LANGUAGE,"Dutch")) AND (LIMIT-TO (AFFILCOUNTRY,"Netherlands")) AND (LIMIT-TO (SUBJAREA,"PSYC") OR LIMIT-TO (SUBJAREA,"SOCI") OR LIMIT-TO (SUBJAREA,"ARTS") OR LIMIT-TO (SUBJAREA,"MULT"))

WorldCat (May 2, 2026)

Ab("expectation" OR "trend" OR "direction" OR "approach" OR "attitude" OR "view" OR "transition" OR "shift" OR "perspective") AND Ti("mental health*" OR "mental wellbeing" OR "psychological" OR "psychiatry") AND Ti("dutch" OR "Netherland*") AND (yr:2023)
Held By Library TU Delft checked

Ab("narrative" OR "discourse") AND Ti("mental health*" OR "mental wellbeing" OR "psychological" OR "psychiatry") AND Ti("dutch" OR "Netherland*") AND (yr:2018)
Held By Library TU Delft checked

PubMed (May 2, 2026)

((("expectation"[Title/Abstract] OR "trend"[Title/Abstract] OR "direction"[Title/Abstract] OR "approach"[Title/Abstract] OR "attitude"[Title/Abstract] OR "view"[Title/Abstract] OR "transition"[Title/Abstract] OR "shift"[Title/Abstract] OR "perspective"[Title/Abstract]) AND ("mental health*"[Title/Abstract] OR "mental wellbeing"[Title/Abstract] OR "psychological"[Title/Abstract] OR "psychiatry"[Title/Abstract]) AND ("dutch"[Title/Abstract] OR "Netherland*"[Title/Abstract]))) AND

(("2023"[Date - Publication] : "3000"[Date - Publication])) AND (Dutch[Language] OR English[Language])

((("discourse"[Title/Abstract]) AND ("mental health*"[Title/Abstract] OR "mental wellbeing"[Title/Abstract] OR "psychological"[Title/Abstract] OR "psychiatry"[Title/Abstract]) AND ("dutch"[Title/Abstract] OR "Netherland*"[Title/Abstract]))) AND (("2018"[Date - Publication] : "3000"[Date - Publication])) AND (Dutch[Language] OR English[Language])

SUPPLEMENTARY MATERIALS 2: INTERVIEW GUIDE

Introduction and consent

Part 1: Society

1. How do you see the Dutch society has changed in its perspective on and approach towards mental healthcare?
2. What indications are there for this transition (e.g. initiatives, money flows)?
3. What is the current context?
4. What do citizens currently expect from a mental healthcare professional?
5. Where are we as a Dutch society going to, regarding the approach towards mental healthcare?
6. Where should we desire to go to?

Part 2: Healthcare system

1. What is the current approach of the healthcare system towards mental health? What do you see in your everyday work field?
2. How was this in the past? What has changed?
3. How do healthcare professionals approach healthy vs. sick, stigma, the relationship with the patient etc.?
4. What should the role of a (mental) healthcare professional be in your opinion?
5. What will become an important skill for mental healthcare professionals?

Part 3: Education

1. If interviewee has been educated in medicine: What is your experience? What have you missed?
2. Regarding the aspects of change you just mentioned, what should the role of education be?
3. What is missing in medicine education to move in the right direction?
4. Which aspects or themes should get more attention in medicine education?
5. Is it a problem of knowledge or skills, and why?
6. What could the role of experts-by-experience be in education?
7. What would you want to say now to a medicine student, what does (s)he need to take with him/her from education?

Wrap up

SUPPLEMENTARY MATERIALS 3: DISTRIBUTION OF CODES IN ALL SOURCES

* 1: Treating a classification, 2: Understanding the patient's problem, 3: Recovering life-balance, 4: Building collective resilience

Table 1. Distribution in reviewed literature.

**Source description**			**Coding results**				
**Author**	**Type**	**Keywords**	**# text units**	**Timeframe**	**Code**	**Theme**	**Narrative***
Albers, Roeg, Nijssen, van Weeghel & Bongers [1]	Quantitative study	Mental illness, discrimination, victimization, perpetration, social functioning, quality of life	4	present, future	collaborate, normal vs not normal, promoting positive mental health, social context and situation	abnormality that can be classified, holistic approach, temporal and contextual distress, work together	1, 3, 4
Alrouh, Pool, Middeldorp & Bartels [2]	Quantitative study	Child, genetic architecture, mental health, twins	4	present	interaction of physical and psychological, normal vs not normal, promoting positive mental health	abnormality that can be classified, holistic approach, imbalance in a complex human	1, 3
Bansema, Vermeiren, Nijland, de Soet, Roelevel, van Ewijk & Nooteboom [3]	Qualitative study	Severity, long term nature of mental health problems, interrelated mental health problems, child and adolescent psychiatry, qualitative research	21	past, present, future	being a classification, embed in society, embracing complexity, engaging with system dynamics, include the patient experience, personal process, problem of individual, seeing the patient as a whole person, segregated and specialized care, social context and situation, symptoms and diseases	abnormality that can be classified, build personal and societal resilience, classification-specific treatment, collective health & acceptation, holistic approach, individual who needs help, take the patient perspective, temporal and contextual distress, therapeutic relationship	1, 2, 3, 4
Beenackers, Kruize, Barsties, Acda, Bakker, Droomers, Kamphuis, Koomen, Nijkamp,	Qualitative study	Population density, inequality, mental health, complex systems approach, group model building	17	present, future	act as a coach, addressing existential aspects, creating collective strength, forming an emotional connection, open the dialogue, personal process, promoting positive mental health, relational	abnormality that can be classified, collective health & acceptation, holistic approach, therapeutic relationship, work together	1, 2, 3, 4

Source description			Coding results				
Author	Type	Keywords	# text units	Timeframe	Code	Theme	Narrative*
Vaandrager, Völker, Luijben & Ruijsbroek [4]					acceptance, symptoms and diseases		
Beckers & Koopmans [5]	Qualitative study	Recovery college, peer worker, personal recovery	10	past, present, future	embed in society, embracing complexity, engaging with system dynamics, social context and situation	build personal and societal resilience, collective health & acceptation, temporal and contextual distress	4
Bergamin, Luigjes, Kiverstein, Bockting & Denys [6]	Review	Autonomy, authenticity, competence, mental illness, self, identity	3	present, future	addressing existential aspects, promoting positive mental health, solve the problem	holistic approach, provide the right treatment	1, 3
Bloem [7]	Historical study	Phenomenology, phenomenological psychiatry, anthropology, anthropological psychiatry, time-experience, inter-war period	8	past	act as a coach, addressing existential aspects, addressing the brain and biology, broaden your skillset, engaging with system dynamics, mental health spectrum, seeing the patient as a whole person	build personal and societal resilience, classification-specific treatment, collective health & acceptation, holistic approach, temporal and contextual distress, therapeutic relationship, work together	1, 2, 3, 4
Boersma & Brown [8]	Qualitative study	Burnout, Habermas, illness labels, legitimacy, metaphor, Parsons, sick role, the Netherlands	4	present, future	addressing existential aspects, being a classification	abnormality that can be classified, holistic approach	1, 3
Bohlmeijer & Westerhof [9]	Review	Positive psychology, mental health care, intervention, implementation model, integration	8	past, present	lack of wellbeing and meaning, promoting positive mental health, seeing the patient as a whole person, symptoms and diseases	abnormality that can be classified, holistic approach, imbalance in a complex human, therapeutic relationship	1, 2, 3
Bozhar, de Rooij, Lok, Vrijkotte,	Quantitative study	Adolescents, discrimination, mental health, social safety,	9	past, present, future	creating collective strength, embed in society, engaging with system dynamics,	build personal and societal resilience, collective health &	3, 4

Source description			Coding results				
**Author**	**Type**	**Keywords**	**# text units**	**Timeframe**	**Code**	**Theme**	**Narrative***
Wiers & Larsen [10]		substance use, urban neighborhood			interaction of physical and psychological, relational acceptance, social context and situation	acceptation, imbalance in a complex human, temporal and contextual distress	
Braun, Lusky, Ben Yehuda & Fruchter [11]	Review	PTSD, veterans, rehabilitation; stigma, peer support, biopsychosocial-spiritual, Israel, health policy	11	present, future	addressing existential aspects, applying protocolized treatment, embed in society, embracing complexity, lack of wellbeing and meaning, problem of individual, promoting positive mental health, relational acceptance, using the impact of relationships	build personal and societal resilience, classification-specific treatment, collective health & acceptation, holistic approach, imbalance in a complex human, individual who needs help, therapeutic relationship	1, 2, 3, 4
Buntinx [12]	In memoriam		11	past, present, future	act as a coach, being a classification, engaging with system dynamics, normal vs not normal, open the dialogue, promoting positive mental health, relational acceptance, symptoms and diseases, using the impact of relationships	abnormality that can be classified, collective health & acceptation, holistic approach, therapeutic relationship, work together	1, 2, 3, 4
Crombach, Janssen, Daemen, Klaassen, van Amelsvoort, Leijdesdorff [13]	Qualitative study	Youth mental health, Access to care, Outreach, Attitudes towards mental health	9	present, future	addressing existential aspects, creating collective strength, embed in society, forming an emotional connection, personal process, relational acceptance	build personal and societal resilience, collective health & acceptation, holistic approach, therapeutic relationship	2, 3, 4
de Jong & Strikwerda [14]	Historical study	Culture of control, disneyisation, precautionary criminal law, prevention, risk society, urban crisis	3	present	normal vs not normal	abnormality that can be classified	1

Source description			Coding results				
**Author**	**Type**	**Keywords**	**# text units**	**Timeframe**	**Code**	**Theme**	**Narrative***
Desai, Panchal, Vala, Ratnani, Jahangirali, Vadher & Khania [15]	Quantitative study	Clinical posting, psychiatry, perspective toward mental illness, undergraduate medical students	2	present, future	explore yourself, segregated and specialized care	build personal and societal resilience, classification-specific treatment	1, 4
Fusar-Poli, de Pablo, de Micheli, Nieman, Corell, Kessing, Pfennig, Bechdolf, Borgwardt & Arango [16]	Scoping review	Good mental health, health promotion, public health, interventions, evidence-based medicine, prevention	17	present, future	act as a coach, addressing existential aspects, engaging with system dynamics, interaction of physical and psychological, lack of wellbeing and meaning, not fitting the social norm, promoting positive mental health, relational acceptance, social context and situation, symptoms and diseases	abnormality that can be classified, collective health & acceptation, holistic approach, imbalance in a complex human, individual who needs help, temporal and contextual distress, work together	1, 2, 3, 4
Godthelp, Heerings, Köhne, Noordhof, Truijens, Nooteboom, Welten [17]	Qualitative study	Child psychiatry, adolescent psychiatry, family therapy, systemic therapy, narrative therapy, recovery-oriented care, trauma-informed care, resilience, realist evaluation, program theory	25	past, present, future	act as a coach, addressing existential aspects, addressing the brain and biology, broaden your skillset, creating collective strength, embracing complexity, engaging with system dynamics, open the dialogue, relational acceptance, using the impact of relationships	build personal and societal resilience, classification-specific treatment, collective health & acceptation, holistic approach, therapeutic relationship, work together	1, 2, 3, 4
Heilig, MacKillop, Martinez, Rehm, Leggio & Vanderschuren [18]	Review	Addiction	5	past, present	addressing the brain and biology, problem of individual, symptoms and diseases	abnormality that can be classified, classification-specific treatment, individual who needs help	1, 2
Hirdes, van Everdingen, Ferris, Franco-	Review	Care planning, outcomes, quality, case-mix,	5	present, future	creating collective strength, engaging with system dynamics, segregated and	abnormality that can be classified, classification-specific treatment,	1, 4

Source description			Coding results				
**Author**	**Type**	**Keywords**	**# text units**	**Timeframe**	**Code**	**Theme**	**Narrative***
Martin, Fries, Heikkilä, Hirdes, Hoffman, James & Martin [19]		psychometric properties, homelessness, integration			specialized care, symptoms and diseases	collective health & acceptation	
Huijs, Braam, Kruizinga, Jacobs, Reijnders & Simons [20]	Quantitative study	Spirituality, well-being; psychopathology, meaning, prospective research	4	past, present	lack of wellbeing and meaning, mental health spectrum, promoting positive mental health	holistic approach, imbalance in a complex human, temporal and contextual distress	3, 4
Huth, van der Wal, Zavlis, Luigjes, Lakerveld, Galenkamp, Lok, Stronks, Bockting, Marsman, Goudriaan, van Holst [21]	Quantitative study	Depression, socio-economic position, ethnicity, network analysis, urban, multi-level, HELIUS study	8	past, present	interaction of physical and psychological, problem of individual, social context and situation	imbalance in a complex human, individual who needs help, temporal and contextual distress	2, 3, 4
Janković, Sijtsema, Bogaerts [22]	Quantitative study	Psychological resilience, social-ecological resilience, mental health issues Social and leisure activities	5	past, present, future	creating collective strength, promoting positive mental health	collective health & acceptation, holistic approach	3, 4
Karbouniaris, Wilken, Weerman & Abma [23]	Qualitative study	Health care professionals' lived experience, experiential knowledge, service users' perceptions, trauma informed care, recovery	14	present, future	addressing existential aspects, distantiate from patient, explore yourself, reveal own vulnerability, using the impact of relationships	build personal and societal resilience, holistic approach, provide the right treatment, therapeutic relationship	1, 2, 3, 4
Kotera, van Laethem, Ohshima [24]	Quantitative study	Cross-culture, Japanese workers, Dutch workers, work mental health, self-compassion, work engagement	5	present, future	addressing existential aspects, mental health spectrum, promoting positive mental health, social context and situation	holistic approach, temporal and contextual distress	3, 4
Kroon, Alma, Bak, van der Krieke, Bruggeman [25]	Qualitative study	Collaboration, Ecosystem, Mental health care	5	present, future	broaden your skillset, collaborate, embracing complexity, include the	abnormality that can be classified, build personal and societal resilience,	1, 2, 3, 4

Source description			Coding results				
**Author**	**Type**	**Keywords**	**# text units**	**Timeframe**	**Code**	**Theme**	**Narrative***
		system, Qualitative study, Transition			patient experience, symptoms and diseases	collective health & acceptation, take the patient perspective, work together	
Lases, Bruins, Scheepers, van Sambeek, Ng, Rennick-Egglestone, Slade, van Balkom & Castelein [26]	Qualitative study	Autism; stories, psychosis, severe mental illness, rehabilitation	12	present, future	addressing existential aspects, being a classification, include the patient experience, promoting positive mental health, relational acceptance, using the impact of relationships	abnormality that can be classified, collective health & acceptation, holistic approach, take the patient perspective, therapeutic relationship	1, 2, 3, 4
Leemrijse, Hujala, van Wezel, Makkes, Bitter, Oksman, Kuronen, Korevaar & Rijken [27]	Conference abstract	Vulnerable populations, multiple problems, primary care, social care, local integration	5	present, future	collaborate, embed in society, segregated and specialized care	build personal and societal resilience, classification-specific treatment, work together	1, 3, 4
Lorenz-Artz, Bierbooms & Bongers [28]	Qualitative study	Peer-supported open dialogue, mental health care, severe mental illness, transformation, network-oriented approach, recovery-based approach, client-centeredness	23	present, future	act as a coach, addressing existential aspects, broaden your skillset, collaborate, creating collective strength, explore yourself, forming an emotional connection, open the dialogue, promoting positive mental health, reveal own vulnerability, social context and situation, solve the problem, using the impact of relationships	build personal and societal resilience, collective health & acceptation, holistic approach, provide the right treatment, temporal and contextual distress, therapeutic relationship, work together	1, 2, 3, 4
Magnée, de Beurs, Schellevis & Verhaak [29]	Dissertation	Basic mental health care, primary care, mental health care, general practitioners, substitution	7	past, present	collaborate, mental health spectrum, segregated and specialized care, solve the problem	classification-specific treatment, provide the right treatment,	1, 3, 4

Source description			Coding results				
**Author**	**Type**	**Keywords**	**# text units**	**Timeframe**	**Code**	**Theme**	**Narrative***
						temporal and contextual distress, work together	
Meerman, Dewinter, Boer, Noot, van der Klink, Glas [30]	Qualitative study	Capability approach, autism, neurodiversity, autistic flourishing, capabilities, wellbeing	6	past, present, future	embed in society, engaging with system dynamics, mental health spectrum, normal vs not normal, promoting positive mental health, symptoms and diseases	abnormality that can be classified, build personal and societal resilience, collective health & acceptation, holistic approach, temporal and contextual distress	1, 3, 4
Nauta, Boenink, Wimalaratne, Menkes, Mellsop & Broekman [31]	Quantitative study	Perspectives toward psychosocial and psychiatric problems in general hospital, chronic drinking problems, dementia, depression, hospital consultants, treatment non-compliance	5	present, future	interaction of physical and psychological, segregated and specialized care, using the impact of relationships	classification-specific treatment, imbalance in a complex human, therapeutic relationship	1, 2, 3
Nooteboom, Kuiper, Mulder, Roetman, Eilander & Vermeiren [32]	Qualitative study	Families, integrated care, mental health, parents, shared decision making	21	present, future	act as a coach, be understandable, collaborate, creating collective strength, embed in society, engaging with system dynamics, open the dialogue, personal process, social context and situation, solve the problem	build personal and societal resilience, collective health & acceptation, holistic approach, provide the right treatment, take the patient perspective, temporal and contextual distress, work together	1, 2, 3, 4
Oosterhuis & Aan de Stegge [33]	Historical study	Coercion, Dutch psychiatry, emotional work, mental nursing, sexuality, suicide	15	past, present, future	be understandable, distantiate from patient, explore yourself, gather medical knowledge, include the patient experience, normal vs not normal, reveal own vulnerability, seeing the patient as a whole person	abnormality that can be classified, build personal and societal resilience, provide the right treatment, take the patient perspective, therapeutic relationship	1, 2, 4

Source description			Coding results				
**Author**	**Type**	**Keywords**	**# text units**	**Timeframe**	**Code**	**Theme**	**Narrative***
Ouwehand [34]	Review	Bipolar disorder, psychopathology, religious experiences, explanatory models, religion, spirituality	9	present, future	acknowledge the pt, addressing existential aspects, collaborate, explore yourself, mental health spectrum, normal vs not normal	abnormality that can be classified, build personal and societal resilience, holistic approach, take the patient perspective, temporal and contextual distress, work together	1, 2, 3, 4
Piot, Attoe, Billon, Cross, Rethans & Falissard [35]	Review	Education medical, learning, mental health, patient simulation, simulation training	5	present	forming an emotional connection, include the patient experience, mental health spectrum, seeing the patient as a whole person, social context and situation	take the patient perspective, temporal and contextual distress, therapeutic relationship	2, 4
Schotanus-Dijkstra, Keyes, de Graaf & ten Have [36]	Quantitative study	Anxiety disorder, longitudinal survey, mental health recovery, mental well-being, mood disorder	2	future	lack of wellbeing and meaning, promoting positive mental health	holistic approach, imbalance in a complex human	3
Schout & de Jong [37]	Review	Family group conferencing, Sloterdijk's sphere theory, kin ties, life-world led interventions, social embeddedness, thick and thin solidarity	5	present, future	creating collective strength, problem of individual	collective health & acceptation, individual who needs help	2, 4
Sempertegui Vallejo, Knipscheer & Bekker [38]	Quantitative study	Competence, depression, diversity, mental health, Moroccan, training, Turkish	8	present, future	applying protocolized treatment, broaden your skillset, embed in society, engaging with system dynamics	build personal and societal resilience, classification-specific treatment, collective health & acceptation	1, 4
Silvius, Antezana & Ghane [39]	Mixed-method study	Cultural formulation interview, cultural competences, disparity,	7	past, present, future	acknowledge the pt, being a classification, engaging with system dynamics, include the	abnormality that can be classified, collective health & acceptation, take the patient	1, 2, 4

Source description			Coding results				
**Author**	**Type**	**Keywords**	**# text units**	**Timeframe**	**Code**	**Theme**	**Narrative***
		inequity, mental health, cultural assessment			patient experience, seeing the patient as a whole person	perspective, therapeutic relationship	
Slimmen, Timmermans, Lechner & Oenema [40]	Quantitative study	Mental wellbeing, social environmental factors, social cohesion, community integration, social structures, social support, social network satisfaction, socioecological model, students, multi-level approach	10	present, future	creating collective strength, embracing complexity, engaging with system dynamics, include the patient experience, relational acceptance, social context and situation	collective health & acceptation, take the patient perspective, temporal and contextual distress	2, 4
Smits, Bakker, Neuteboom, van Rosmalen, Hoop & de Loos [41]	Conference abstract	Resident based, innovative working method, integrated care ecosystem	1	future	act as a coach	work together	3
Snijders, Pries, Sgammeglia, Al Jowf, Youssef, de Nijs, Guloksuz & Rutten [42]	Review	Prospective longitudinal studies, resilience, resilience-promoting interventions, review, stress	3	future	addressing the brain and biology, lack of wellbeing and meaning, relational acceptance	classification-specific treatment, collective health & acceptation, imbalance in a complex human	1, 3, 4
Stolper, van Doesum, Henselmans, Bijl & Steketee [43]	Qualitative study	Integrated family approach, parental mental disorder, adult and child mental health services, infants and early childhood, transmission of psychopathology, family focused practice, qualitative study	8	present, future	applying protocolized treatment, collaborate, creating collective strength, embed in society, engaging with system dynamics, problem of individual	build personal and societal resilience, classification-specific treatment, collective health & acceptation, individual who needs help, work together	1, 2, 3, 4
‘t Lam, Vingerhoets & Bylsma [44]	Quantitative study	Crying, emotions, psychotherapy, tears, therapists	8	present, future	distantiate from patient, forming an emotional connection, reveal own	build personal and societal resilience, provide the right	1, 2, 4

Source description			Coding results				
**Author**	**Type**	**Keywords**	**# text units**	**Timeframe**	**Code**	**Theme**	**Narrative***
					vulnerability, using the impact of relationships	treatment, therapeutic relationship	
van de Beek, Landman, Veling, Schoevers & van der Krieke [45]	Qualitative study	Cultural factors, explanations for mental health problems, mental health, qualitative research, social determinants, transcultural psychiatry	10	present	addressing existential aspects, addressing the brain and biology, embed in society, problem of individual, social context and situation	build personal and societal resilience, classification-specific treatment, holistic approach, individual who needs help, temporal and contextual distress	1, 2, 3, 4
van den Broek, de Vroege, Metz, Gribling, de Ridder & van Eerd [46]	Review		9	past, present, future	act as a coach, addressing existential aspects, applying protocolized treatment, embed in society, open the dialogue	build personal and societal resilience, classification-specific treatment, holistic approach, work together	1, 3, 4
van Grootheest, Vahl, Ensink, Koop, Zegwaard, Hein & Middeldorp [47]	Qualitative study		6	present, future	creating collective strength, embed in society, embracing complexity, problem of individual	build personal and societal resilience, collective health & acceptation, individual who needs help	2, 4
van Heteren, Raaphorst, Groeneveld & Bussemaker [48]	Qualitative study	Concept formation, culture, health, health personnel, interview, professionalism, methods, qualitative methods, social workers	10	present, future	acknowledge the pt, forming an emotional connection, include the patient experience, interaction of physical and psychological, not fitting the social norm, problem of individual, promoting positive mental health, seeing the patient as a whole person, social context and situation, symptoms and diseases	abnormality that can be classified, holistic approach, imbalance in a complex human, individual who needs help, take the patient perspective, temporal and contextual distress, therapeutic relationship	1, 2, 3, 4
van Langen, Szöke, Rijkelijkhuizen,	Qualitative study	Attention Deficit Hyperactivity Disorder,	18	present	addressing the brain and biology, embracing complexity, mental health	abnormality that can be classified, classification-specific treatment,	1, 2, 4

Source description			Coding results				
**Author**	**Type**	**Keywords**	**# text units**	**Timeframe**	**Code**	**Theme**	**Narrative***
Durston, van Hulst [49]		ADHD, psychoeducation, discourse analysis			spectrum, normal vs not normal, not fitting the social norm, solve the problem, symptoms and diseases	collective health & acceptation, individual who needs help, provide the right treatment, temporal and contextual distress	
van Os, Guloksuz, Vijn, Hafkenscheid & Delespaul [50]	Review	Mental health care, evidence-based practice, relational components of care, public health, resilience, peer support, trans-syndromal symptom reduction, recovery, e-communities	26	past, present, future	addressing existential aspects, addressing the brain and biology, applying protocolized treatment, being a classification, broaden your skillset, collaborate, creating collective strength, embracing complexity, engaging with system dynamics, lack of wellbeing and meaning, mental health spectrum, segregated and specialized care, symptoms and diseases, using the impact of relationships	abnormality that can be classified, build personal and societal resilience, classification-specific treatment, collective health & acceptation, holistic approach, imbalance in a complex human, temporal and contextual distress, therapeutic relationship, work together	1, 2, 3, 4
van Os, Scheepers, Milo, Ockeloen, Guloksuz & Delespaul [51]	Review	Public mental health, mental health services, social care, recovery college, mental health, mental health reform, social trials	47	past, present, future	acknowledge the pt, act as a coach, addressing existential aspects, addressing the brain and biology, broaden your skillset, creating collective strength, distantiate from patient, embed in society, engaging with system dynamics, gather medical knowledge, mental health spectrum, open the dialogue, personal process, promoting positive mental health,	abnormality that can be classified, build personal and societal resilience, classification-specific treatment, collective health & acceptation, holistic approach, provide the right treatment, take the patient perspective, temporal and contextual distress, work together	1, 2, 3, 4

Source description			Coding results				
**Author**	**Type**	**Keywords**	**# text units**	**Timeframe**	**Code**	**Theme**	**Narrative***
					relational acceptance, reveal own vulnerability, social context and situation, solve the problem, symptoms and diseases		
van Sambeek, Franssen, van Geelen & Scheepers [52]	Qualitative study	Trauma, psychosis, meaning-making, narrative identity, service-user perspective, personal recovery, stigma, narrative analysis	16	past, present, future	acknowledge the pt, addressing existential aspects, broaden your skillset, explore yourself, forming an emotional connection, lack of wellbeing and meaning, open the dialogue, personal process, relational acceptance, social context and situation	build personal and societal resilience, collective health & acceptation, holistic approach, imbalance in a complex human, take the patient perspective, temporal and contextual distress, therapeutic relationship, work together	2, 3, 4
van Steden [53]	Qualitative study	Law enforcement, mental illness, public health, street-level professionals	4	present, future	engaging with system dynamics, mental health spectrum, normal vs not normal, problem of individual	abnormality that can be classified, collective health & acceptation, individual who needs help, temporal and contextual distress	1, 2, 4
van Vuuren, Uitenbroek, van der Wal & Chinapaw [54]	Quantitative study	Adolescence, epidemiology, mental health, sociodemographic factors, time trends	4	present	mental health spectrum, not fitting the social norm	individual who needs help, temporal and contextual distress	2, 4
Wiers & Verschure [55]	Review	Addiction, brain disease model, neurorehabilitation, systems approach	6	past, present, future	addressing the brain and biology, engaging with system dynamics, problem of individual, seeing the patient as a whole person	classification-specific treatment, collective health & acceptation, individual who needs help, therapeutic relationship	1, 2, 4

Table 2. Distribution in semi-structured interviews.

Interviewee	Nr. of extracts	Timeframe	Code	Theme	Narrative*
1	9	present, future	collaborate, embed in society, forming an emotional connection, open the dialogue, symptoms and diseases	abnormality that can be classified, build personal and societal resilience, therapeutic relationship, work together	1, 2, 3, 4
2	17	present, future	acknowledge the pt, broaden your skillset, collaborate, distantiate from patient, explore yourself, forming an emotional connection, include the patient experience, not fitting the social norm, seeing the patient as a whole person, symptoms and diseases	abnormality that can be classified, build personal and societal resilience, individual who needs help, provide the right treatment, take the patient perspective, therapeutic relationship, work together	1, 2, 3, 4
3	28	present, future	acknowledge the pt, act as a coach, applying protocolized treatment, being a classification, collaborate, distantiate from patient, engaging with system dynamics, explore yourself, forming an emotional connection, include the patient experience, mental health spectrum, normal vs not normal, not fitting the social norm, promoting positive mental health, reveal own vulnerability, solve the problem	abnormality that can be classified, build personal and societal resilience, classification-specific treatment, collective health & acceptation, holistic approach, individual who needs help, provide the right treatment, take the patient perspective, temporal and contextual distress, therapeutic relationship, work together	1, 2, 3, 4
4	21	present, future	acknowledge the pt, being a classification, collaborate, distantiate from patient, engaging with system dynamics, explore yourself, include the patient experience, not fitting the social norm, reveal own vulnerability	abnormality that can be classified, build personal and societal resilience, collective health & acceptation, individual who needs help, provide the right treatment, take the patient perspective, work together	1, 2, 3, 4
5	20	present, future	act as a coach, addressing existential aspects, collaborate, explore yourself, forming an emotional connection, gather medical knowledge, include the patient experience, reveal own vulnerability, using the impact of relationships	build personal and societal resilience, holistic approach, provide the right treatment, take the patient perspective, therapeutic relationship, work together	1, 2, 3, 4
6	40	past, present, future	acknowledge the pt, act as a coach, addressing the brain and biology, being a classification, collaborate, creating collective strength, embed in society, embracing complexity, engaging with system dynamics, explore yourself, forming an emotional connection, include the patient experience, normal vs not normal, not fitting the social norm, open the dialogue, personal process, problem of individual, relational acceptance,	abnormality that can be classified, build personal and societal resilience, classification-specific treatment, collective health & acceptation, holistic approach, individual who needs help, provide the right treatment, take the patient	1, 2, 3, 4

Interviewee	Nr. of extracts	Timeframe	Code	Theme	Narrative*
			reveal own vulnerability, seeing the patient as a whole person, social context and situation, solve the problem	perspective, temporal and contextual distress, therapeutic relationship, work together	
7	40	past, present, future	act as a coach, addressing existential aspects, addressing the brain and biology, applying protocolized treatment, being a classification, creating collective strength, distantiate from patient, embed in society, engaging with system dynamics, explore yourself, include the patient experience, mental health spectrum, open the dialogue, problem of individual, segregated and specialized care, solve the problem	abnormality that can be classified, build personal and societal resilience, classification-specific treatment, collective health & acceptation, holistic approach, individual who needs help, provide the right treatment, take the patient perspective, temporal and contextual distress, work together	1, 2, 3, 4
8	20	present, future	acknowledge the pt, addressing existential aspects, creating collective strength, distantiate from patient, embed in society, explore yourself, forming an emotional connection, gather medical knowledge, include the patient experience, mental health spectrum, seeing the patient as a whole person, segregated and specialized care, solve the problem	build personal and societal resilience, classification-specific treatment, collective health & acceptation, holistic approach, provide the right treatment, take the patient perspective, temporal and contextual distress, therapeutic relationship	1, 2, 3, 4
9	30	past, present, future	act as a coach, addressing the brain and biology, applying protocolized treatment, being a classification, creating collective strength, embed in society, embracing complexity, explore yourself, gather medical knowledge, include the patient experience, problem of individual, promoting positive mental health, seeing the patient as a whole person, symptoms and diseases	abnormality that can be classified, build personal and societal resilience, classification-specific treatment, collective health & acceptation, holistic approach, individual who needs help, provide the right treatment, take the patient perspective, therapeutic relationship, work together	1, 2, 3, 4
10	20	past, present, future	act as a coach, broaden your skillset, explore yourself, gather medical knowledge, interaction of physical and psychological, mental health spectrum, not fitting the social norm, seeing the patient as a whole person, social context and situation, solve the problem	build personal and societal resilience, imbalance in a complex human, individual who needs help, provide the right treatment, temporal and contextual distress, therapeutic relationship, work together	1, 2, 3, 4
11	26	past, present, future	addressing existential aspects, being a classification, collaborate, creating collective strength, explore yourself, forming an emotional connection, include the patient experience, normal vs not normal, not fitting the	abnormality that can be classified, build personal and societal resilience, collective health & acceptation, holistic approach,	1, 2, 3, 4

Interviewee	Nr. of extracts	Timeframe	Code	Theme	Narrative*
			social norm, problem of individual, promoting positive mental health, seeing the patient as a whole person, social context and situation, solve the problem, using the impact of relationships	individual who needs help, provide the right treatment, take the patient perspective, temporal and contextual distress, therapeutic relationship, work together	

Table 3. Distribution in interviews from podcast episodes.

Episode	Nr. of extracts	Timeframe	Code	Theme	Narrative*
1	8	present, future	addressing existential aspects, broaden your skillset, collaborate, open the dialogue, seeing the patient as a whole person, using the impact of relationships	build personal and societal resilience, holistic approach, therapeutic relationship, work together	2, 3, 4
2	3	present	being a classification, solve the problem	abnormality that can be classified, provide the right treatment	1
3	2	future	explore yourself, social context and situation	build personal and societal resilience, temporal and contextual distress	4
4	9	future	act as a coach, addressing existential aspects, broaden your skillset, collaborate, open the dialogue, reveal own vulnerability, using the impact of relationships	build personal and societal resilience, holistic approach, therapeutic relationship, work together	2, 3, 4
5	2	present	being a classification, not fitting the social norm	abnormality that can be classified, individual who needs help	1, 2
6	3	present, future	being a classification, broaden your skillset, seeing the patient as a whole person	abnormality that can be classified, build personal and societal resilience, therapeutic relationship	1, 2, 4
7	8	present, future	act as a coach, addressing existential aspects, collaborate, forming an emotional connection, reveal own vulnerability	build personal and societal resilience, holistic approach, therapeutic relationship, work together	2, 3, 4
8	7	present, future	being a classification, mental health spectrum, normal vs not normal, seeing the patient as a whole person	abnormality that can be classified, temporal and contextual distress, therapeutic relationship	1, 2, 4
9	2	future	addressing existential aspects, engaging with system dynamics	collective health & acceptation, holistic approach	3, 4

Episode	Nr. of extracts	Timeframe	Code	Theme	Narrative*
10	11	past, present, future	applying protocolized treatment, be understandable, being a classification, creating collective strength, engaging with system dynamics, mental health spectrum, open the dialogue, social context and situation, symptoms and diseases, using the impact of relationships	abnormality that can be classified, classification-specific treatment, collective health & acceptation, take the patient perspective, temporal and contextual distress, therapeutic relationship, work together	1, 2, 3, 4

SUPPLEMENTARY MATERIALS 4: REPORTING GUIDELINES CHECKLISTS

SUPPLEMENTARY MATERIALS 4: REPORTING GUIDELINES CHECKLISTS

COREQ (COnsolidated criteria for REporting Qualitative research) Checklist

Topic	Item No.	Guide Questions/Description	Reported on Page No.
**Domain 1: Research team and reflexivity**			
Personal characteristics			
Interviewer/facilitator	1	Which author/s conducted the interview or focus group?	5
Credentials	2	What were the researcher's credentials? E.g. PhD, MD	5
Occupation	3	What was their occupation at the time of the study?	5
Gender	4	Was the researcher male or female?	5
Experience and training	5	What experience or training did the researcher have?	5
Relationship with participants			
Relationship established	6	Was a relationship established prior to study commencement?	5
Participant knowledge of the interviewer	7	What did the participants know about the researcher? e.g. personal goals, reasons for doing the research	N/A
Interviewer characteristics	8	What characteristics were reported about the inter viewer/facilitator? e.g. Bias, assumptions, reasons and interests in the research topic	N/A
**Domain 2: Study design**			
Theoretical framework			
Methodological orientation and Theory	9	What methodological orientation was stated to underpin the study? e.g. grounded theory, discourse analysis, ethnography, phenomenology, content analysis	4, 6
Participant selection			
Sampling	10	How were participants selected? e.g. purposive, convenience, consecutive, snowball	5
Method of approach	11	How were participants approached? e.g. face-to-face, telephone, mail, email	5
Sample size	12	How many participants were in the study?	5
Non-participation	13	How many people refused to participate or dropped out? Reasons?	5
Setting			
Setting of data collection	14	Where was the data collected? e.g. home, clinic, workplace	5
Presence of non-participants	15	Was anyone else present besides the participants and researchers?	5
Description of sample	16	What are the important characteristics of the sample? e.g. demographic data, date	5, 6
Data collection			
Interview guide	17	Were questions, prompts, guides provided by the authors? Was it pilot tested?	Supplementar
Repeat interviews	18	Were repeat inter views carried out? If yes, how many?	N/A
Audio/visual recording	19	Did the research use audio or visual recording to collect the data?	5
Field notes	20	Were field notes made during and/or after the inter view or focus group?	5
Duration	21	What was the duration of the inter views or focus group?	5
Data saturation	22	Was data saturation discussed?	N/A
Transcripts returned	23	Were transcripts returned to participants for comment and/or	N/A

Topic	Item No.	Guide Questions/Description	Reported on Page No.
		correction?	
**Domain 3: analysis and findings**			
Data analysis			
Number of data coders	24	How many data coders coded the data?	6, 7
Description of the coding tree	25	Did authors provide a description of the coding tree?	6, 7, Data avail
Derivation of themes	26	Were themes identified in advance or derived from the data?	6
Software	27	What software, if applicable, was used to manage the data?	6
Participant checking	28	Did participants provide feedback on the findings?	N/A
Reporting			
Quotations presented	29	Were participant quotations presented to illustrate the themes/findings? Was each quotation identified? e.g. participant number	11
Data and findings consistent	30	Was there consistency between the data presented and the findings?	8-11
Clarity of major themes	31	Were major themes clearly presented in the findings?	8-11
Clarity of minor themes	32	Is there a description of diverse cases or discussion of minor themes?	8-11

Developed from: Tong A, Sainsbury P, Craig J. Consolidated criteria for reporting qualitative research (COREQ): a 32-item checklist for interviews and focus groups. *International Journal for Quality in Health Care*. 2007. Volume 19, Number 6: pp. 349 – 357

Once you have completed this checklist, please save a copy and upload it as part of your submission. DO NOT include this checklist as part of the main manuscript document. It must be uploaded as a separate file.

Preferred Reporting Items for Systematic reviews and Meta-Analyses extension for Scoping Reviews (PRISMA-ScR) Checklist

SECTION	ITEM	PRISMA-ScR CHECKLIST ITEM	REPORTED ON PAGE #
**TITLE**			
Title	1	Identify the report as a scoping review.	1
**ABSTRACT**			
Structured summary	2	Provide a structured summary that includes (as applicable): background, objectives, eligibility criteria, sources of evidence, charting methods, results, and conclusions that relate to the review questions and objectives.	2
**INTRODUCTION**			
Rationale	3	Describe the rationale for the review in the context of what is already known. Explain why the review questions/objectives lend themselves to a scoping review approach.	3
Objectives	4	Provide an explicit statement of the questions and objectives being addressed with reference to their key elements (e.g., population or participants, concepts, and context) or other relevant key elements used to conceptualize the review questions and/or objectives.	4
**METHODS**			
Protocol and registration	5	Indicate whether a review protocol exists; state if and where it can be accessed (e.g., a Web address); and if available, provide registration information, including the registration number.	N/A
Eligibility criteria	6	Specify characteristics of the sources of evidence used as eligibility criteria (e.g., years considered, language, and publication status), and provide a rationale.	4
Information sources*	7	Describe all information sources in the search (e.g., databases with dates of coverage and contact with authors to identify additional sources), as well as the date the most recent search was executed.	4
Search	8	Present the full electronic search strategy for at least 1 database, including any limits used, such that it could be repeated.	Supplementary material
Selection of sources of evidence†	9	State the process for selecting sources of evidence (i.e., screening and eligibility) included in the scoping review.	4, 5
Data charting process‡	10	Describe the methods of charting data from the included sources of evidence (e.g., calibrated forms or forms that have been tested by the team before their use, and whether data charting was done independently or in duplicate) and any processes for obtaining and confirming data from investigators.	6
Data items	11	List and define all variables for which data were sought and any assumptions and simplifications made.	6
Critical appraisal of individual sources of evidence§	12	If done, provide a rationale for conducting a critical appraisal of included sources of evidence; describe the methods used and how this information was used in any data synthesis (if appropriate).	N/A

St. Michael's
Inspired Care.
Inspiring Science.

SECTION	ITEM	PRISMA-ScR CHECKLIST ITEM	REPORTED ON PAGE #
Synthesis of results	13	Describe the methods of handling and summarizing the data that were charted.	6
**RESULTS**			
Selection of sources of evidence	14	Give numbers of sources of evidence screened, assessed for eligibility, and included in the review, with reasons for exclusions at each stage, ideally using a flow diagram.	7
Characteristics of sources of evidence	15	For each source of evidence, present characteristics for which data were charted and provide the citations.	Supplementary material
Critical appraisal within sources of evidence	16	If done, present data on critical appraisal of included sources of evidence (see item 12).	N/A
Results of individual sources of evidence	17	For each included source of evidence, present the relevant data that were charted that relate to the review questions and objectives.	Data availability
Synthesis of results	18	Summarize and/or present the charting results as they relate to the review questions and objectives.	7-12
**DISCUSSION**			
Summary of evidence	19	Summarize the main results (including an overview of concepts, themes, and types of evidence available), link to the review questions and objectives, and consider the relevance to key groups.	12, 13
Limitations	20	Discuss the limitations of the scoping review process.	14, 15
Conclusions	21	Provide a general interpretation of the results with respect to the review questions and objectives, as well as potential implications and/or next steps.	15
**FUNDING**			
Funding	22	Describe sources of funding for the included sources of evidence, as well as sources of funding for the scoping review. Describe the role of the funders of the scoping review.	15

JBI = Joanna Briggs Institute; PRISMA-ScR = Preferred Reporting Items for Systematic reviews and Meta-Analyses extension for Scoping Reviews.

* Where *sources of evidence* are compiled from, such as bibliographic databases, social media platforms, and sites.

† A more inclusive/heterogeneous term used to account for the different types of evidence or data sources (e.g., quantitative and/or qualitative research, expert opinion, and policy documents) that may be eligible in a scoping review as opposed to only studies. This is not to be confused with *information sources* (see first footnote).

‡ The frameworks by Arksey and O'Malley (6) and Levac and colleagues (7) and the JBI guidance (4, 5) refer to the process of data extraction in a scoping review as data charting.

§ The process of systematically examining research evidence to assess its validity, results, and relevance before using it to inform a decision. This term is used for items 12 and 19 instead of "risk of bias" (which is more applicable to systematic reviews of interventions) to include and acknowledge the various sources of evidence that may be used in a scoping review (e.g., quantitative and/or qualitative research, expert opinion, and policy document).

From: Tricco AC, Lillie E, Zarin W, O'Brien KK, Colquhoun H, Levac D, et al. PRISMA Extension for Scoping Reviews (PRISMAScR): Checklist and Explanation. Ann Intern Med. 2018;169:467–473. doi: 10.7326/M18-0850.

REFERENCES

1. Albers WM, Roeg DP, Nijssen Y, Van Weeghel J, Bongers IM. Profiling of victimization, perpetration, and participation: a latent class analysis among people with severe mental illness. Plos one 2018;13(11):e0208457. doi: 10.1371/journal.pone.0208457
2. Alrouh H, Pool R, Middeldorp C, Bartels M. Enduring Mental Health in Childhood and Adolescence: Prevalence, Prediction, and Genetic Architecture. Journal of the American Academy of Child & Adolescent Psychiatry 2026;65(4):539-51. doi: 10.1016/j.jaac.2025.05.001
3. Bansema CH, Vermeiren RRJM, Nijland L, de Soet R, Roeleveld J, van Ewijk H, et al. Towards identifying the characteristics of youth with severe and enduring mental health problems in practice: a qualitative study. European Child & Adolescent Psychiatry 2024;33(7):2365-75. doi: 10.1007/s00787-023-02325-2
4. Beenackers MA, Kruize H, Barsties L, Acda A, Bakker I, Droomers M, et al. Urban densification in the Netherlands and its impact on mental health: An expert-based causal loop diagram. Health & Place 2024;87:103218. doi: 10.1016/j.healthplace.2024.103218
5. Beckers T, Koopmans M. Recovery colleges as enablers of personal recovery: qualitative evaluation of the development of a recovery college in the Netherlands. BMC Psychiatry 2025;25(1):465. doi: 10.1186/s12888-025-06917-x
6. Bergamin J, Luigjes J, Kiverstein J, Bockting CL, Denys D. Defining autonomy in psychiatry. Frontiers in Psychiatry 2022;13:801415. doi: 10.3389/fpsyt.2022.801415
7. Bloem S. Hoe 'tijd' psychiatrie opnieuw betoverde [How ‘time’ enchanted psychiatry again]. Studium 2018;10(4). doi: 10.18352/studium.10154
8. Boersma JJ, Brown P. The tired hero and her (il) legitimation: Reworking Parsons to analyse experiences of burnout within the Dutch employment system and lifeworld. Social Science & Medicine 2020;265:113471. doi: 10.1016/j.socscimed.2020.113471
9. Bohlmeijer E, Westerhof G. The model for sustainable mental health: future directions for integrating positive psychology into mental health care. Frontiers in psychology 2021;12:747999. doi: 10.3389/fpsyg.2021.747999
10. Bozhar H, de Rooij SR, Lok A, Vrijkotte TGM, Wiers RW, Larsen H. The Role of Discrimination in Social Safety and its Interplay with Adolescents’ Mental Health and Substance Use: A Network Perspective. Journal of Youth and Adolescence 2026. doi: 10.1007/s10964-026-02344-7
11. Braun D, Lusky M, Ben Yehuda Y, Fruchter E. Western Models of PTSD Rehabilitation Among Military Veterans: A Narrative Comparative Review and Policy Implications for Israel. Healthcare 2026;14(7):929. doi: 10.3390/healthcare14070929
12. Buntinx W. Paradigma-verschuiving in de visie op zorg voor mensen met een verstandelijke handicap [Paradigm-shift in the vision on care for people with an intellectual disability] 2020.
13. Crombach CMW, Janssen SER, Daemen M, Klaassen RMC, van Amelsvoort T, Leijdesdorff SMJ. Everybody @ease - reaching Out To Vulnerable Subgroups of Young People with Mental Health Problems in the Netherlands: an Adaptation of the Standard @ease Working Method. Child Psychiatry & Human Development 2025. doi: 10.1007/s10578-025-01861-z
14. de Jong W, Strikwerda L. Controlling risks in the safe city: The rise of pre-emptive practices in law enforcement, public surveillance and mental health and addiction care (1970–2020). Urban Studies 2021;58(12):2514-30. doi: 10.1177/0042098020952669
15. Desai R, Panchal B, Vala A, Ratnani IJ, Vadher S, Khania P. Impact of clinical posting in psychiatry on the attitudes towards psychiatry and mental illness in undergraduate medical students. General Psychiatry 2019;32(3). doi: 10.1136/gpsych-2019-100072
16. Fusar-Poli P, de Pablo GS, De Micheli A, Nieman DH, Correll CU, Kessing LV, et al. What is good mental health? A scoping review. European Neuropsychopharmacology 2020;31:33-46. doi: 10.1016/j.euroneuro.2019.12.105
17. Godthelp J, Heerings M, Köhne A, Noordhof A, Truijens F, Nooteboom L, et al. Addressing the complexity of recovery beyond reductionist, diagnosis-driven care: a realist evaluation of a family-

centered, narrative-based child and adolescent psychiatry practice. BMC Psychiatry 2026. doi: 10.1186/s12888-026-07933-1
18. Heilig M, MacKillop J, Martinez D, Rehm J, Leggio L, Vanderschuren LJ. Addiction as a brain disease revised: why it still matters, and the need for consilience. Neuropsychopharmacology 2021;46(10):1715-23. doi: 10.1038/s41386-020-00950-y
19. Hirdes JP, van Everdingen C, Ferris J, Franco-Martin M, Fries BE, Heikkilä J, et al. The interRAI suite of mental health assessment instruments: an integrated system for the continuum of care. Frontiers in psychiatry 2020;10:926. doi: 10.3389/fpsyt.2019.00926
20. Huijs T, Braam AW, Kruizinga R, Jacobs N, Reijnders J, Simons M. Spirituality as a Predictor of Well-Being, Mental Distress or Both: A Four-Week Follow-Up Study in a Sample of Dutch and Belgian Adults. Religions 2024;15(2):179. doi: 10.3390/rel15020179
21. Huth KBS, van der Wal J, Zavlis O, Luigjes J, Lakerveld J, Galenkamp H, et al. Individual and neighborhood determinants of depressive symptoms in ethnic minorities in the urban HELIUS sample: a multi-level network perspective. Social Science & Medicine 2025;381:118195. doi: 10.1016/j.socscimed.2025.118195
22. Janković M, Sijtsema J, Bogaerts S. A Network Analysis of Social and Leisure Activities, Resilience, and Mental Health: Differences by Adverse Childhood Experiences. Journal of Happiness Studies 2026;27(2):36. doi: 10.1007/s10902-025-01001-3
23. Karbouniaris S, Wilken JP, Weerman A, Abma T. Experiential knowledge of mental health professionals. Service users' perceptions. European Journal of Mental Health 2022;17(3):23-37. doi: 10.5708/EJMH.17.2022.3.2
24. Kotera Y, Van Laethem M, Ohshima R. Cross-cultural comparison of mental health between Japanese and Dutch workers: Relationships with mental health shame, self-compassion, work engagement and motivation. Cross Cultural & Strategic Management 2020;27(3):511-30. doi: 10.1108/CCSM-02-2020-0055
25. Kroon SJC, van der Krieke L, Bruggeman R, Alma MA. The uneven triad: a qualitative study of perspectives of relatives, patients, and professionals on (not) involving relatives in mental health. BMC Psychiatry 2025;25(1):384. doi: 10.1186/s12888-025-06814-3
26. Lases MN, Bruins J, Scheepers FE, van Sambeek N, Ng F, Rennick-Egglestone S, et al. Is personal recovery a transdiagnostic concept? Testing the fit of the CHIME framework using narrative experiences. Journal of Mental Health 2025;34(3):254-62. doi: 10.1080/09638237.2024.2361225
27. Leemrijse C, Hujala A, van Wezel P, Makkes N, Bitter N, Oksman E, et al. Providing integrated health and social care to vulnerable populations in the community. International Journal of Integrated Care (IJIC) 2018;18:51. doi: 10.5334/ijic.s2051
28. Lorenz-Artz K, Bierbooms J, Bongers I. Introducing Peer-supported Open Dialogue in changing mental health care. Frontiers in Psychology 2023;13:1056071. doi: 10.3389/fpsyg.2022.1056071
29. Magnée T, de Beurs D, Schellevis F, Verhaak P. Developments in mental health care in Dutch general practices: an overview of recent studies. Tijdschrift Voor Psychiatrie 2019;61(2):126-34.
30. Meerman J, Dewinter J, Boer J, Noot K, van der Klink J, Glas G. Identification of Capabilities of Autistic Young Adults: Towards an Understanding of Autistic Flourishing. Child Psychiatry & Human Development 2025. doi: 10.1007/s10578-025-01935-y
31. Nauta K, Boenink A, Wimalaratne I, Menkes D, Mellsop G, Broekman B. Attitudes of general hospital consultants towards psychosocial and psychiatric problems in Netherlands. Psychology, health & medicine 2019;24(4):402-13. doi: 10.1080/13548506.2018.1546020
32. Nooteboom LA, Kuiper CH, Mulder EA, Roetman PJ, Eilander J, Vermeiren RR. What do parents expect in the 21st century? A qualitative analysis of integrated youth care. International Journal of Integrated Care 2020;20(3):8. doi: 10.5334/ijic.5419
33. Oosterhuis H, Aan de Stegge C. Between Emotional Involvement and Professional Detachment: The Challenges of Nursing in Dutch Mental Institutions (1880–1980). Social History of Medicine 2021;34(4):1277-396. doi: 10.1093/shm/hkaa086

34. Ouwehand E. Religious Experiences in the Context of Bipolar Disorder: Serious Pathology and/or Genuine Spirituality? A Narrative Review against the Background of the Literature about Bipolar Disorder and Religion. Religions 2024;15(3):274. doi: 10.3390/rel15030274
35. Piot M-A, Attoe C, Billon G, Cross S, Rethans J-J, Falissard B. Simulation training in psychiatry for medical education: a review. Frontiers in Psychiatry 2021;12:658967. doi: 10.3389/fpsyt.2021.658967
36. Schotanus-Dijkstra M, Keyes CL, de Graaf R, Ten Have M. Recovery from mood and anxiety disorders: The influence of positive mental health. Journal of affective disorders 2019;252:107-13. doi: 10.1016/j.jad.2019.04.051
37. Schout G, De Jong G. The weakening of kin ties: Exploring the need for life-world led interventions. International Journal of Environmental Research and Public Health 2018;15(2):203. doi: 10.3390/ijerph15020203
38. Sempertegui GA, Knipscheer JW, Bekker MH. Development and evaluation of diversity-oriented competence training for the treatment of depressive disorders. Transcultural psychiatry 2018;55(1):31-54. doi: 10.1177/1363461517725224
39. Silvius L, Antezana J KV, Ghane S. Symptom vs context: lessons learned from a large-scale implementation of the Cultural Formulation Interview. Frontiers in Psychiatry 2024;15. doi: 10.3389/fpsyt.2024.1410865
40. Slimmen S, Timmermans O, Lechner L, Oenema A. The direct and indirect effects of social environmental factors on student mental wellbeing at different socio-ecological levels: A longitudinal perspective. Wellbeing, Space and Society 2025;9:100294. doi: 10.1016/j.wss.2025.100294
41. Smits S, Bakker B, Neuteboom J, van Rosmalen F, Hoop L, de Loos S. Experiment Ruwaard: Thinking, acting and organizing differently! International Journal of Integrated Care (IJIC) 2018;18:28. doi: 10.5334/ijic.s2028
42. Snijders C, Pries L-K, Sgammeglia N, Al Jowf G, Youssef NA, de Nijs L, et al. Resilience against traumatic stress: current developments and future directions. Frontiers in psychiatry 2018;9:676. doi: 10.3389/fpsyt.2018.00676
43. Stolper H, van Doesum K, Henselmans P, Bijl AL, Steketee M. The Patient's Voice as a Parent in Mental Health Care: A Qualitative Study. International Journal of Environmental Research and Public Health 2022;19(20):13164. doi: 10.3390/ijerph192013164
44. 't Lam C, Vingerhoets A, Bylsma L. Tears in therapy: A pilot study about experiences and perceptions of therapist and client crying. European Journal of Psychotherapy & Counselling 2018;20(2):199-219. doi: 10.1080/13642537.2018.1459767
45. van de Beek MH, Landman E, Veling W, Schoevers RA, van der Krieke L. Discussing the unspoken: A qualitative analysis of online forum discussions on mental health problems in young Moroccan-Dutch migrants. Transcultural psychiatry 2023;60(1):86-98. doi: 10.1177/13634615221105118
46. van den Broek A, de Vroege L, Metz M, Gribling G, de Ridder A, van Eerd J. Aanpassing aan veranderende zorgbehoefte in de ggz: mentale gezondheidscentra [Adaptation to a changing care need in mental healthcare: mental health centres]. Tijdschrift Voor Psychiatrie 2022;64(2):101-7.
47. van Grootheest R, Vahl P, Koop F, Zegwaard A, Heln I, Middeldorp C. Family-focused approach in mental health care, an overview of the current offer in the Netherlands. Tijdscrift voor Psychiatrie 2025;67(9):504-9.
48. van Heteren F, Raaphorst N, Groeneveld S, Bussemaker J. Professionals' health conceptions of clients with psychosocial problems: An analysis based on an empirical exploration of semi-structured interviews. International Journal of Nursing Studies Advances 2023;5:100120. doi: 10.1016/j.ijnsa.2023.100120
49. van Langen MJM, Szőke R, Rijkelijkhuizen DNJ, Durston S, van Hulst BM. Lost in explanation: internal conflicts in the discourse of ADHD psychoeducation. BMC Psychiatry 2022;22(1):690. doi: 10.1186/s12888-022-04327-x

50. van Os J, Guloksuz S, Vijn TW, Hafkenscheid A, Delespaul P. The evidence-based group-level symptom-reduction model as the organizing principle for mental health care: time for change? World Psychiatry 2019;18(1):88-96. doi: 10.1002/wps.20609
51. van Os J, Scheepers F, Milo M, Ockeloen G, Guloksuz S, Delespaul P. "It has to be better, otherwise we will get stuck." A Review of Novel Directions for Mental Health Reform and Introducing Pilot Work in the Netherlands. Clinical Practice & Epidemiology in Mental Health 2023;19:e17450179271206. doi: 10.2174/0117450179271206231114064736
52. van Sambeek N, Franssen G, van Geelen S, Scheepers F. Making meaning of trauma in psychosis. Frontiers in Psychiatry 2023;14. doi: 10.3389/fpsyt.2023.1272683
53. van Steden R. Governing through care: a qualitative assessment of team play between police and nurses for people with mental illness. International journal of law and psychiatry 2020;68:101532. doi: 10.1016/j.ijlp.2019.101532
54. van Vuuren CL, Uitenbroek DG, Van der Wal MF, Chinapaw MJ. Sociodemographic differences in 10-year time trends of emotional and behavioural problems among adolescents attending secondary schools in Amsterdam, The Netherlands. European Child & Adolescent Psychiatry 2018;27:1621-31. doi: 10.1007/s00787-018-1157-5
55. Wiers RW, Verschure P. Curing the broken brain model of addiction: Neurorehabilitation from a systems perspective. Addictive Behaviors 2021;112:106602. doi: 10.1016/j.addbeh.2020.106602
