# Supplementary material for: Mapping narratives on mental health to facilitate collaboration, communication and alignment of care: a systematic scoping review and interview study
Source: BMJ Open. 2026 Jul 16;16(7):e110663. doi: 10.1136/bmjopen-2025-110663 (PMC13384187; doi:10.1136/bmjopen-2025-110663)
Supplement: Supplementary file 2 [file bmjopen-2025-110663.reviewer_comments.pdf]

PEER REVIEW HISTORY

BMJ Open publishes all reviews undertaken for accepted manuscripts. Reviewers are asked to complete a checklist review form and are provided with free text boxes to elaborate on their assessment. These free text comments are reproduced below.

ARTICLE DETAILS

Title (Provisional)

Mapping Narratives on Mental Health to Facilitate Collaboration, Communication and Alignment of Care: A Systematic Scoping Review and Interview Study

Authors

Spek, Deanne; Ermers, Marieke; Rietjens, Judith; Vegt, Niko; Milota, Megan M

VERSION 1 - REVIEW

**Reviewer**	**1**
**Name**	**Bellass, Sue**
**Affiliation**	**Newcastle University, Faculty of Medical Sciences,**
**Date**	**28-Oct-2025**
**COI**	**None**

Thank you for the opportunity to review this interesting manuscript, which is clear and concise, with helpful use of tables to present the data and your analysis. I believe the paper represents an important contribution to the knowledge base and recommend it for publication, but major revisions are needed to address the limitations identified. My first comment relates to your analytic approach. There is insufficient discussion of the

challenges represented by analysing primary and secondary data, and I would like more detail on your approach, and, given there are different conceptualisations of triangulation, what you understand the process to achieve. You state that the findings were consistent across all sources, which, given the variety, seems quite surprising. If the reader were assured that you had rigorously explored the sources, guided by a

methodological framework or triangulation protocol e,g, convergence/ complementarity / dissonance / silence, that would support credibility of the findings.

More detail is needed on the scoping review. The figures in the 'identification' section at the top of the PRISMA flow chart are incorrect: (443 + 423) - 132 = 734, not 759 as

reported in the screening stage. Also, you mentioned searching in Worldcat, but no results are reported from those searches. I would also like to know why the words 'narrative' and 'discourse' weren't used as search terms, when the former at least is a key component of

your research questions. Although recognised elements of the scoping review approach, you should also state why you took such an inclusive approach to the sources and why you did not reject articles on the basis of methodological quality.

Regarding your empirical work, I wondered how you identified people for interview who deviated from the master narratives, when you had not yet articulated what the narratives were. I also would have liked more critical discussion of the podcasts, including any methodological and ethical considerations around use of those data, and the selection of people invited to speak on the podcast. Regarding the findings, it felt a little artificial to present all the themes and then go on to talk about the evolution of the themes over time. Is there a way to integrate the evolution into the theme description, rather than presenting it separately? In table 4 (page 13) I also wondered whether there was an option of presenting proportions rather than numbers.

Thank you again for the opportunity to review this paper. I have also noticed a few typos or queries about word choice, please see below:

Page 3 line 28 - to *make* these changes explicit

Page 4 line 35 - a change *in* how

Page 5 line 5 - do you mean evolvement or evolution?

Page 6 line 27 - there is an error message Page 6 line 54 - 'on the work floor' isn't an expression I'm familiar with

Page 8 line 57 - use of the word 'fundament' in the table heading could perhaps be replaced by 'summary'

Page 10 line 37 - 'making warm contact' should be reworded

Page 11 line 38 - do you mean acceptation or acceptance?

Page 11 line 57 - the word 'bearing' doesn't quite seem to fit here

Page 14 line 57 - delete 'have'

Page 15 line 12 - 'zoom in' is a little colloquial.

**Reviewer**	**2**
**Name**	**Todowede, Olamide**
**Affiliation**	**University of Nottingham**
**Date**	**08-Apr-2026**
**COI**	**None**

This is a well-written manuscript that provides more information about the mental health narrative and how it has evolved among the Dutch population.

I suggest that the author work on the following.

1. Work on the title to inform readers early that this is from a mental healthcare professional's perspective, including in the Abstract.

2. I would have wanted to know about past narratives of mental health within the Dutch population, which would have been helpful in the Introduction and also in the discussion section and that will present what was and what is evolving now, which is currently missing in the manuscript

3. Was there a reason that people with lived experience of a mental health condition were not included in the semi-structured interview?

4. In the Method section, please state earlier what your search period is. I only read that in the discussion section. Please present how the different methods inform each other at the end of the method section

5. The participant who was in the podcast and interview, is that not double-counting? Was their input treated as one? I assume they will say the same thing. Please include how this was treated in the analysis, rather than just mentioning that they are the same.

6. The discussion section needs to be reworked, what your study found, what the literature has said about this and what has changed.

VERSION 1 - AUTHOR RESPONSE

	**Reviewer 1**	
1	My first comment relates to your analytic approach. There is insufficient discussion of the challenges represented by analysing primary and secondary data, and I would like more detail on your approach, and, given there are different conceptualisations of triangulation, what you understand the process to achieve. You state that the findings were consistent across all sources, which, given the variety, seems quite surprising. If the reader were assured that you had rigorously explored the sources, guided by a methodological framework or triangulation protocol e,g, convergence/ complementarity / dissonance / silence, that would support credibility of the findings.	We employed investigator, data source and methodological triangulation, with two reviewers integrating data from three different sources captured via three different methods. The comment of the reviewer led us to the work of Farmer et al (2006), which supported us in further specifying our argumatation for the use of triangulation in our research, and in describing what approach we have used. The approach was connected to specific challenges represented by analysing primary and secondary data, most importantly:<br>-To avoid privileging richness of interview and podcast data, one author (NV) did not listen to the interviews or podcasts but started with the scoping review.<br>-To address the fact that literary sources had already gone through interpretation of the authors and provided more explicit indications of master narratives, particular attention was given to the coding of interview and podcast data.<br>-Analysis of data extracted from each source was combined to capture the totally of master narratives present. Only after completing the analysis, the representation of the various

		narratives across the different sources was analyzed.<br><br>*Changes in manuscript*<br>Methods section, page 8 and 9: we added an explanation regarding our approach to triangulation why and how the different sources were integrated, and how the main triangulation challenges of our study were addressed at the end of the Methods section.<br>Abstract section, Strengths and limitations subsection, page 3: first two bullet points have been integrated to align with the updated description in the Methods section.
2	The figures in the 'identification' section at the top of the PRISMA flow chart are incorrect: (443 + 423) - 132 = 734, not 759 as reported in the screening stage. Also, you mentioned searching in Worldcat, but no results are reported from those searches.	We now present the correct numbers, including the updated search (see comment 5 of reviewer 1).<br><br>*Changes in manuscript:*<br>Methods section, Systematic scoping review subsection, page 5/6: Figure 1 has been updated.
3	I would also like to know why the words 'narrative' and 'discourse' weren't used as search terms, when the former at least is a key component of your research questions.	These words were not used in the initial search, since we did not start our exploration with the aim to find narratives or discourses. We aimed to find a shift in attitudes, approaches, and views. Only after analysis, it became clear that what we had found were collective stories, shared master narratives underlying the shifting attitudes, views and approaches. As suggested by the reviewer, we now included these terms as well. Therefore, we not only updated our search with regards to the timeframe, but we also performed a second search with the terms ‘dicourse’ and ‘narrative’. The search term ‘discourse’ provided 30 records to be screened, ‘narrative’ provided 96 records to be screened. Most records containing the term ‘narrative’ were a narrative synthesis or analysis, often not referring to mental health nor master narratives. From the 126 (30+126) records, 2 were eventually included in the scoping review.<br><br>*Changes in manuscript:*<br>Methods section, Systematic scoping review subsection, paragraph 2, page 5: a sentence explaining the search update has been added.<br>Methods section, Systematic scoping review subsection, paragraph 4, page 5: reasons for exclusion have been elaborated.<br>Methods section, Systematic scoping review subsection, page 5/6: Figure 1 has been updated.<br>Results section, page 9-14: has been adapted to integrate the new search updates.<br>Supplementary materials 1: two additional search strings for the updated search with regards to time (2023-2026) and search terms (discourse and narrative in the ful period of 2018-2026) have been added.

		Supplementary materials 2: has been adapted to integrate the new search updates.
4	Although recognised elements of the scoping review approach, you should also state why you took such an inclusive approach to the sources and why you did not reject articles on the basis of methodological quality.	We did not do a quality appraisal since this study aims to capture master narratives in a wide range of resources even including podcast interviews.<br><br>*Changes in manuscript*<br>Methods section, Systematic scoping review subsection, paragraph 3, page 6: sentence explaining our rationale for not performing a quality appraisal has been added.
5	Regarding your empirical work, I wondered how you identified people for interview who deviated from the master narratives, when you had not yet articulated what the narratives were.	Interviewees were identified by asking various mental health and educational staff members at the hospital to name colleagues whose understanding of and approach to mental health differed from the perceived standard or norm.<br><br>*Changes in manuscript*<br>Methods section, Semi-structured interviews subsection, paragraph 2, page 6: a sentence explaining how we identified interviews has been added.
6	I also would have liked more critical discussion of the podcasts, including any methodological and ethical considerations around use of those data, and the selection of people invited to speak on the podcast.	We used podcasts as they are deemed representative for public views and provide access to a wide variety of perspectives which could not be included in our own interviews.<br>Methodologically, the podcast selection was based on the selection criteria outlined by Kulkov et al (2024). They also recommend thematic analysis for data analysis.<br>Since the selected podcast episodes are openly available on multiple platforms to the public, we deemed it unnecessary to ask all podcast interviewees for consent. We did ask the creator of the podcast for consent after showing her how we had used and referred to her podcast episodes.<br><br>*Changes in manuscript*<br>Methods section, Podcast interviews, paragraph 1, page 7: methodological and ethical considerations around use of podcasts as data have been added.
7	Regarding the findings, it felt a little artificial to present all the themes and then go on to talk about the evolution of the themes over time. Is there a way to integrate the evolution into the theme description, rather than presenting it separately?	After careful consideration, we decided to keep the segments seperately from the narrative descriptions. Our decision to divide the 'Results' section as we have helps support the structure of our research questions (question 1 and 2) and approach for data analysis (thematic and content analysis). We thereby think the current structure will support comprehension of the findings for most readers.
8	In table 4 (page 13) I also wondered whether there was an option of presenting proportions rather than numbers.	Based on the suggestion of the reviewer, we decided to show percentages instead of stating the number of sources.<br><br>*Changes in manuscript*

		Results section, Shifting emphasis on narratives subsection, page 14: percentages instead of numbers have been included in Table 4.
9	I have also noticed a few typos or queries about word choice, please see below:<br><br>Page 3 line 28 - to *make* these changes explicit<br>Page 4 line 35 - a change *in* how<br>Page 5 line 5 - do you mean evolvement or evolution?<br>Page 6 line 27 - there is an error message Page 6 line 54 - 'on the work floor' isn't an expression I'm familiar with<br>Page 8 line 57 - use of the word 'fundament' in the table heading could perhaps be replaced by 'summary'<br>Page 10 line 37 - 'making warm contact' should be reworded<br>Page 11 line 38 - do you mean acceptation or acceptance?<br>Page 11 line 57 - the word 'bearing' doesn't quite seem to fit here<br>Page 14 line 57 - delete 'have'<br>Page 15 line 12 - 'zoom in' is a little colloquial.	We corrected the typos kindly identified by the reviewer.<br><br>Regarding the reviewer’s comment on evolvement or evulotion, we now decided to use the word ‘shift’ and at the same time emphasize that this shift does not mean a replacement but a change in nuance and sometimes growing coexistence of master narratives. We have chosen this wording because we want to describe an ongoing change that doesn’t necessarily effect how everyone sees mental health in general. In other words, we try to capture the presence of a *shift* in emphasis or dominance of certain views and attitudes.<br><br>*Changes in manuscript*<br>Lines indicated by reviewer.<br>Use of ‘shift’ and nuancing our wording throughout the whole paper, including the abstract.
	**Reviewer 2**	
10	Work on the title to inform readers early that this is from a mental healthcare professional's perspective, including in the Abstract.	We thank the reviewer for noticing the importance of the title. However, the article also includes patients’ perspectives as decsribed in the Limtations section. Hence we decided to keep the title as it was. To prevent misunderstanding by future readers, we adjusted the section on Patient and public involvement.<br>Based on the comment of the reviewer, we now address the underrepresentaion of patient and relative perspectives in the abstract as well.<br><br>*Changes in manuscript*<br>Patient and public involvement section, page 19: the description in this section has been further clarified.<br>Abstract section, Conclusions subsection, page 2: the necessity for more research including patients’ and relatives’ perspectives has been added.
11	I would have wanted to know about past narratives of mental health within the Dutch population, which would have been helpful in the Introduction and also in the discussion section and that will present what was and what is evolving now, which is currently missing in the manuscript	We now offer a brief summary of past narratives in the Introduction. It is not possible to provide a full historical overview as this is beyond the scope of this manuscript. Since our goal is to move to recommendations and support for the future, we did not discuss them in the Discussion.<br><br>*Changes in manuscript*<br>Introduction Section, paragraph 4: a summary of past narratives and references to find more information has been added.

12	Was there a reason that people with lived experience of a mental health condition were not included in the semi-structured interview?	Yes, as already explained in the section on Patient and public involvement (page 18) we unfortunately had no resources for interviewing this target group. That being said, one of the professionals working at the hospital was an expert-by-experience. The limited patient perspective is one of the reasons we combined our interview data with the scoping review and the podcast; patient perspectives are represented in these two sources.<br><br>*Changes in manuscript*<br>Patient and public involvement section, page 19: the description in this section was further clarified.
13	In the Method section, please state earlier what your search period is. I only read that in the discussion section.	We think the reviewer might have missed the search period mention in the Method section (subsection Systematic scoping review). To make this search period more prominent, we now mention it as the first inclusion criterium instead of the last.<br><br>*Changes in manuscript*<br>Methods section, Systematic scoping review subsection, paragraph 3, page 5: order of inclusion criteria has been changed.
14	Please present how the different methods inform each other at the end of the method section.	We provided further clarification of how the different methods inform each other at the end of the Methods section.<br><br>*Changes in manuscript*<br>Methods section, page 8 and 9: a description of how different methods informed each other has been added.
15	The participant who was in the podcast and interview, is that not double-counting? Was their input treated as one? I assume they will say the same thing. Please include how this was treated in the analysis, rather than just mentioning that they are the same.	The participant was included in the semi-structured interviews even though this participant was already part of the podcast. The podcast focussed mainly on how mental healthcare is changing; we were also interested in current narratives and in implications for medical education. The podcast data and semi-structured interview data from this person were treated as two separate sources to rightfully represent what narratives were brought forward by podcast data versus semi-structured interview data. The podcast interview had taken place two years prior to the semi-structured interview and the view of this participant may thus have changed.<br><br>*Changes in manuscript*<br>Methods section, Podcast interviews subsection, paragraph 2, page 8: rationale for interviewing a participant who was part of the podcast as well as procedure of treating this data has been added.

16	The discussion section needs to be reworked, what your study found, what the literature has said about this and what has changed.	We have now reworked the discussion section in line with the suggestions of the reviewer.<br><br>*Changes in manuscript*<br>Discussion section, paragraph 2, page 15: a paragraph on what the literature has said about our findings and what is different from our findings has been added.<br>Introduction section, paragraph 2, page 3: an explanation about the fact that most literature addresses discourses and not master narratives has been added.
